# Core binding factor (CBF) is required for Epstein-Barr virus EBNA3 proteins to regulate target gene expression

**DOI:** 10.1093/nar/gkw1167

**Published:** 2016-11-28

**Authors:** Kostas Paschos, Quentin Bazot, Guiyi Ho, Gillian A. Parker, Jonathan Lees, Geraint Barton, Martin J. Allday

**Affiliations:** 1 Molecular Virology, Department of Medicine, Imperial College London, Norfolk Place, London, W2 1PG, UK; 2 Institute of Structural and Molecular Biology, Division of Biosciences, University College London, Gower Street, London WC1E 6BT, UK; 3 Centre for Integrative Systems Biology and Bioinformatics, Imperial College London, London SW7 2AZ, UK

## Abstract

ChIP-seq performed on lymphoblastoid cell lines (LCLs), expressing epitope-tagged EBNA3A, EBNA3B or EBNA3C from EBV-recombinants, revealed important principles of EBNA3 binding to chromatin. When combined with global chromatin looping data, EBNA3-bound loci were found to have a singular character, each directly associating with either EBNA3-repressed or EBNA3-activated genes, but not with both. EBNA3A and EBNA3C showed significant association with repressed and activated genes. Significant direct association for EBNA3B loci could only be shown with EBNA3B-repressed genes. A comparison of EBNA3 binding sites with known transcription factor binding sites in LCL GM12878 revealed substantial co-localization of EBNA3s with RUNX3—a protein induced by EBV during B cell transformation. The beta-subunit of core binding factor (CBFβ), that heterodimerizes with RUNX3, could co-immunoprecipitate robustly EBNA3B and EBNA3C, but only weakly EBNA3A. Depletion of either RUNX3 or CBFβ with lentivirus-delivered shRNA impaired epitope-tagged EBNA3B and EBNA3C binding at multiple regulated gene loci, indicating a requirement for CBF heterodimers in EBNA3 recruitment during target-gene regulation. ShRNA-mediated depletion of CBFβ in an EBNA3C-conditional LCL confirmed the role of CBF in the regulation of EBNA3C-induced and -repressed genes. These results reveal an important role for RUNX3/CBF during B cell transformation and EBV latency that was hitherto unexplored.

## INTRODUCTION

Despite being associated with various cancers—including several B cell lymphomas—Epstein-Barr Virus (EBV) infects, persistently and asymptomatically, >90% of the human population (1,2). The EBV life cycle is closely linked to the normal B cell differentiation pathway (reviewed in 3,4). Infection of mature B cells by EBV initially leads to their activation and differentiation into proliferating B blasts. Latency-associated genes expressed at this stage, termed latency III, encode six EBV nuclear antigen proteins, three latent membrane proteins, two small non-coding RNAs and several microRNAs. The viral nuclear antigens expressed include EBNA3A, EBNA3B and EBNA3C—a family of related, but non-redundant EBV proteins, expressed from three genes arranged in tandem within a complex transcription unit (reviewed in 5). *In vitro*, infected mature B cells give rise to lymphoblastoid cell lines (LCLs) that carry the EBV genome as an extra-chromosomal episome and remain in the latency III state expressing all latency associated EBV genes and resembling cycling, antigen-activated B blasts. The ease of obtaining continuously proliferating LCLs from virtually any genetic background has led to LCLs being used in diverse studies with extensive data being generated on genome-wide transcription factor localization, global chromatin dynamics and analyses of the global epigenetic landscape (see below).

In the context of viral latency, the EBNA3 family of proteins appears to facilitate a fine balance between activation/proliferation and protection from the oncogenic potential this can cause (5,6). They primarily do this by controlling host gene transcription. EBNA3A and EBNA3C together specifically repress, among many other host genes, pro-apoptotic *BIM/BCL2L11* (7) and anti-proliferative *p16^INK4A^* (8–10) two tumor suppressors that would otherwise contribute to an oncogenic stress response resulting from virus-induced cell activation and proliferation (6,11–13). In contrast, EBNA3B has been shown to act as a tumor suppressor in a humanised-mouse model and in human tumors, in part by facilitating immune cell trafficking and T cell surveillance (14).

EBNA3 function in controlling host transcription is well established, with more than a thousand genes probably co-regulated (15). For each EBNA3, microarray experiments revealed hundreds of host genes to be differentially regulated when cells expressing functional or wild type EBNA3s were compared with cells expressing inactivated EBNA3s or infected with EBNA3 knock-out virus (9,15–20).

Although they do not bind to DNA (21), the EBNA3s robustly associate with chromatin and, by chromatin immunoprecipitation (ChIP), they have been found to localise at and around many EBNA3-regulated genes (examples in 19,22–26). Indeed, EBNA3 ChIP-seq experiments have been performed previously and thousands of EBNA3 binding sites have been identified (24,27–29). These studies have been very informative, each an improvement on the previous, as reagents and resources have become available. The current study continues this trend of optimization, with a new recombinant virus constructed to express an N-terminally tagged EBNA3B in an LCL and with the use of a more appropriate background control.

The ChIP-seq data produced were analysed in combination with experimental genome-wide chromatin data, including global chromatin looping data, available for LCL GM12878—a ‘tier 1’ cell line of the ENCODE project (30,31)—and microarray data identifying EBNA3-regulated genes (9,15–20). The analysis offers new insights into the character of EBNA3 binding sites and their relationship with EBNA3-regulated genes.

Further analysis revealed RUNX3 as the transcription factor most significantly co-localizing with each EBNA3. This was indicated in other studies (24,28–29), but here has been explored in detail. RUNX3 is a member of the RUNX family of proteins that have been found to act as oncogenes or tumor suppressors, depending on context and cell type (32,33). Each RUNX (RUNX1, RUNX2, RUNX3) heterodimerizes with core binding factor β subunit (CBFβ) to form core-binding factor (CBF) (34). It is well established that RUNX3 is an important factor during B cell development (35–37). Here, we show that RUNX3-containing CBF is also required for establishing stable EBNA3B and EBNA3C complexes on chromatin and provide direct evidence that this association is necessary for the regulation of selected EBNA3C target genes.

## MATERIALS AND METHODS

### Production of recombinant EBV expressing TAP-tagged EBNA3B

The FLAG-STREP II Tandem Affinity Purification (TAP) tag (38) was fused to the N-terminus of EBNA3B in the B95-8 EBV BAC (39). This was done using a RecA-mediated homologous recombination system described previously (40). Briefly, the TAP-tag sequence (38) was cloned into the pKovKanΔCm shuttle plasmid containing a DNA sequence running from within the end of EBNA3A through to the end of EBNA3B exon 1, resulting in homologous regions to the EBV B95-8 BAC either side of the TAP-tag insert. The TAP tag was inserted in frame immediately after the ATG start codon and is separated from EBNA3B by the peptide linker ASNGGSGEAS. RecA-mediated homologous recombination between the shuttle plasmid and the B95-8 BAC in DH10B *Escherichia coli*, using previously described methods (7,40), generated the required recombinant TAP-3B BAC.

The recombinant BAC was transfected into HEK293 cells, the integrity of the EBV genome was tested by episome rescue and restriction enzyme digestions analysed by pulsed-field gel electrophoresis. Virus was produced and its titer assessed as described previously (9).

### Production of shRNA expressing lentiviruses

Double-stranded DNA for the stem sequence of each shRNA was created by the annealing of single stranded oligos (Supplementary Table S1) and cloned into either pLKO.1 (Addgene plasmid #10878) or Tet-pLKO-puro (Addgene plasmid #21915) based lentiviral plasmids (41). After validation, 10 μg of each construct was co-transfected (calcium phosphate precipitate method) with helper plasmids psPAX2 (8 μg) and pMD2.G (2 μg) into 293T cells in 10cm culture dishes for lentivirus particle production (seeded at 2.5 × 10^6^ on the previous day). Virus-containing medium was collected 48 h post-transfection (∼4 ml in each case).

### Viral infection of cells

Primary B cells were infected with EBV to produce LCLs as described previously (9). For lentiviral infections of LCLs, 8 μg/μl polybrene was added to 20 × 10^6^ cells in 6ml of medium 15 min before infections. Cells were then pelleted by centrifugation, re-suspended in 1 ml of lentivirus-containing 293T medium and centrifuged again at 450 g for 1.5 h at room temperature. The cells were then resuspended in 5 ml of RPMI medium and transferred to flasks. After 48 h, further 6ml RPMI were added, containing puromycin so that final puromycin concentration was 1 μg/ml.

### Cell culture and treatments

LCLs were grown at 37°C in 10% CO_2_. Newly infected cells were kept at 5% CO_2_ for 2 weeks. All cells were cultured in RPMI-1640 medium (Invitrogen), supplemented with 10% foetal bovine serum, penicillin and streptomycin. Puromycin was added at 1 μg/ml when selection was required. The activating ligand 4-hydroxytamoxifen (HT) was added to 400 nM and doxycycline (DOX) to 500 ng/ml, where indicated. These supplements were both added to cultures every time fresh medium was added to the cells (three times per week).

### Immunoprecipitations, western blots and quantification of mRNA

Co-immunoprecipitations and western blots were performed as previously described (42). Supplementary Table S2 is a list of all antibodies used in this study. RNA extraction and mRNA quantification were performed as previously described (42). However, ALAS1 was used as an endogenous control, in addition to GNB2L1, for normalization with both giving similar results (data not shown). Sequences for primers used are shown in Supplementary Table S3.

### Chromatin immunoprecipitations

ChIP for factors other than the EBNA3s was performed as described previously (42). ChIPs for the TAP-tagged EBNA3s were done as above, but with the following modifications. A suspension of 15 × 10^6^ fixed cells was made in 1 ml swelling buffer (25 M HEPES, pH 7.8; 1.5 mM MgCl_2_; 10 mM KCl, 0.1% NP-40; 1 mM DTT; 1 mM PMSF; 1 μg/ml aprotinin; 1 μg/ml pepstatin A) and incubated at 4°C with rotation for 20 min. They were then centrifuged at 375g for 5 min at 4°C and the supernatant discarded. The remaining nuclei were re-suspended in 1ml of sonication buffer (50 mM HEPES pH 7.9; 140 mM NaCl; 1mM EDTA; 1% Triton X-100; 0.1% sodium deoxycholate; 0.1% SDS; 1 mM PMSF; 1 μg/ml aprotinin; 1 μg/ml pepstatin A) and incubated on ice for 30 min. Lysate was then sonicated for 1 h using a Covaris M220 Focused-ultrasonicator with a milliTUBE holder (settings: peak power 75, duty factor 26, cycles/burst 200, set point temperature 6°C). Sonicated lysate was centrifuged at 12 000g for 10 min at 4°C and supernatant was diluted with 3 ml of sonication buffer. From the input sample 5% was kept at 4°C as a control and the rest was incubated overnight with 16 μg of α-FLAG antibody and 120 μl of ChIP-grade protein G magnetic beads (NEB, #9006) at 4°C in a 15 ml Falcon tube on rollers. The following day the beads were washed with the wash buffers described previously (42), 4 ml buffer for each wash for 15 min at 4°C on rollers. Precipitated chromatin was eluted in 400 μl of elution buffer, the eluate was treated with RNase, formaldehyde cross-links were reversed and DNA cleaned as described before (42), for ChIP sample and input control. Sequences for primers used to assess ChIPed DNA are shown in Supplementary Table S3. In order to obtain DNA for subsequent next generation sequencing, the Qiagen MinElute PCR purification kit was used as described in manufacturer's instructions.

### Next generation sequencing

DNA from ChIP was run on a 2% agarose gel (Bio-Rad Low Range Ultra, #161-3107) and DNA between 100–500 bp was excised and purified using the Qiagen MinElute gel purification kit, according to manufacturer's instructions. At least 1 ng of DNA for each sample was then sent to the Harvard Biopolymers facility for library construction (ChIP-Seq Wafergen) and sequencing (Illumina HiSeq 2500, 50 bp single-reads).

### ChIP-Seq data analysis

Sequence reads were mapped to the human genome (vhg19) downloaded from UCSC (43) using BWA (44). Uniquely mapped reads obtained were 22.7 × 10^6^ for 3A-TAP, 69 × 10^6^ for TAP-3B, 25.7 × 10^6^ for 3C-TAP and 34 × 10^6^ for the non-tagged LCL. Binding regions were identified using the MACS (45) peak-calling algorithm comparing the sample to a control sample processed in the same way from an LCL not expressing tagged proteins. Peaks were defined as significant with a *q* value cut-off of 5.00e-02 and are given in Supplementary File 1. Random numbers were generated by Microsoft Excel RANDBETWEEN function. Peak co-localizations were determined using Partek^®^ software, Version 6.6 Copyright, Partek Inc., St. Louis, MO, USA. Co-localization was defined as two peaks with one or more base pairs in common. Publicly available peak and region tracks used are listed in Supplementary File S2 with link addresses. RBPJ data was obtained from (46). RBPJ peaks were called again using MACS as above for hg19, after read alignments provided were converted from hg18 to hg19 version of the human genome using the LiftOver utility on UCSC. A list of EBNA3-regulated genes considered in this study and direction of regulation is given in Supplementary File S3. Co-ordinates of genes were extracted using UCSC Table Browser tool (https://genome.ucsc.edu/cgi-bin/hgTables). Genes were described as within contact domains if at least the transcription start site (TSS) was contained within a domain. Peaks were described as being within a contact domain if at least one base pair was shared. Pearson's chi-squared test was performed using a 2 × 2 contingency table (47) in each case described in the results section. The average length of contact domains not containing regulated genes was found to be <10% different, compared to the average length of contact domains that do contain regulated genes, in each case (EBNA3A, EBNA3B, EBNA3C activated or repressed genes).

## RESULTS

### EBNA3s associate with enhancers, regions flanking active transcription start sites and quiescent regions of the human genome

ChIP-seq was performed to study the localization of EBNA3A, EBNA3B and EBNA3C across the host genome. The LCLs used were created by infecting primary B cells from a single donor with recombinant EBV expressing epitope-tagged EBNA3A, EBNA3B or EBNA3C. EBNA3A and EBNA3C were tagged at the C-terminus (22,42) with a tandem affinity purification (TAP) tag that combines a FLAG epitope with a double Strep II epitope (3A-TAP and 3C-TAP) (38). However, because we and others (29,48) have found that the addition of polypeptides at the C-terminus of EBNA3B leads to a significant decrease in EBNA3C expression and impairment of LCL growth, we created a recombinant EBV with the same TAP tag at the N-terminus (TAP-3B). In TAP-3B LCLs created, EBNA3C levels were not significantly affected, nor were the levels of other EBV latent proteins in these cells, apart from the levels of TAP-3B itself, which were slightly higher than in cells infected with an unmodified (wild type) BAC-derived EBV (Supplementary Figure S1A). Moreover, the cells proliferated at a similar rate to LCLs infected with the wild type, non-tagged virus. The presence of a functional TAP tag fused to expressed EBNA3B was verified by successfully immunoprecipitating TAP-3B with an α-FLAG antibody and detecting it with an antibody specific for EBNA3B (Supplementary Figure S1B).

**Figure 1.**
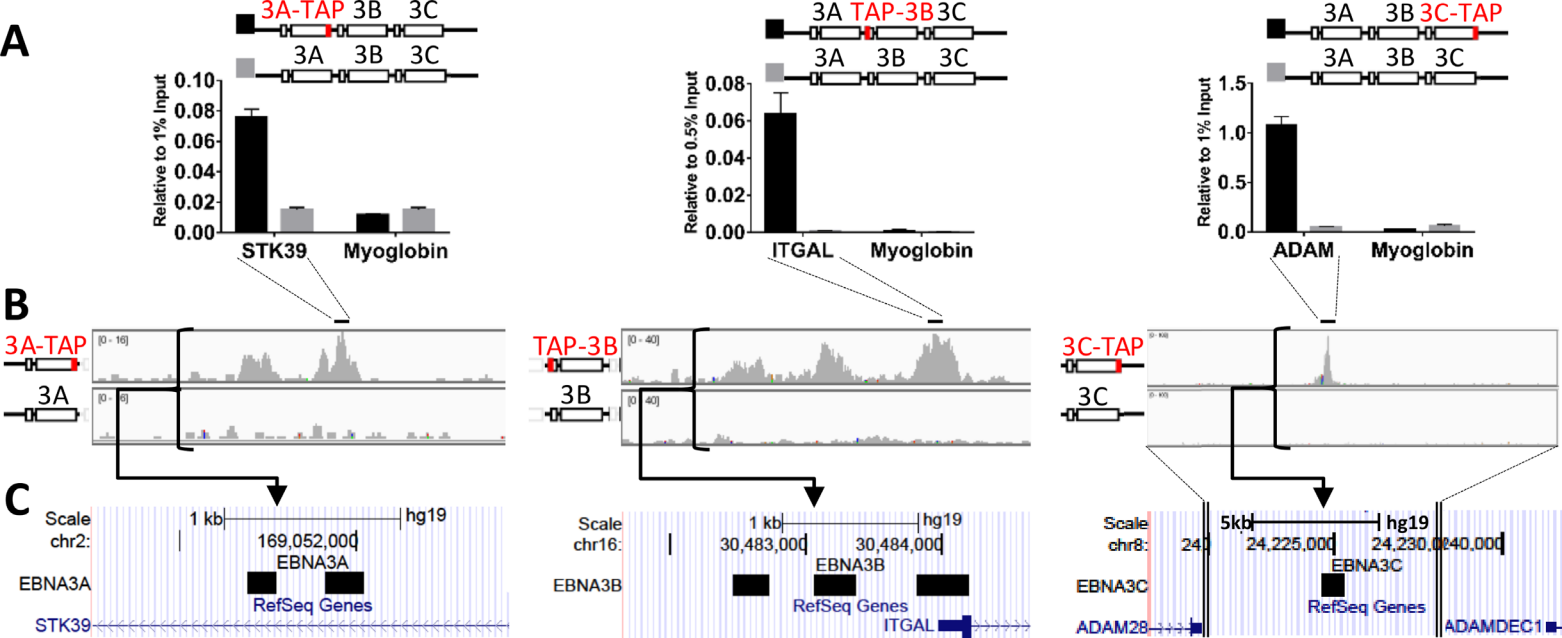
ChIP-seq design and quantification of EBNA3 binding sites on the human genome. (**A**) Multiple ChIP experiments were performed, using an α-FLAG antibody to precipitate EBNA3A- and EBNA3C-tagged with a tandem array purification (TAP) tag at the C-terminus or to precipitate EBNA3B with the same tag fused at the N-terminus (TAP-3B). LCLs expressed each of these proteins from BAC-derived EBV recombinants. For a background control, the same ChIP was performed in an LCL infected with a wild type virus, not expressing tagged proteins. Multiple ChIPed DNA samples from each cell line were pooled and assayed by QPCR for enrichment, relative to input, at known EBNA3 target sites on the genome (17,19,27). The Myoglobin gene promoter region was used as a negative control. Histogram bar heights represent enrichment over percentage of input stated; error bars represent standard deviation between triplicate QPCR runs. (**B**) ChIPed DNA was subjected to next generation sequencing and reads produced were mapped to the human genome (version hg19) using the BWA algorithm (44). (**C**) Using the aligned reads produced from ChIP in non-tagged LCL, 3A-TAP, TAP-3B and 3C-TAP peaks were called using the MACS algorithm (45).

**Figure 2.**
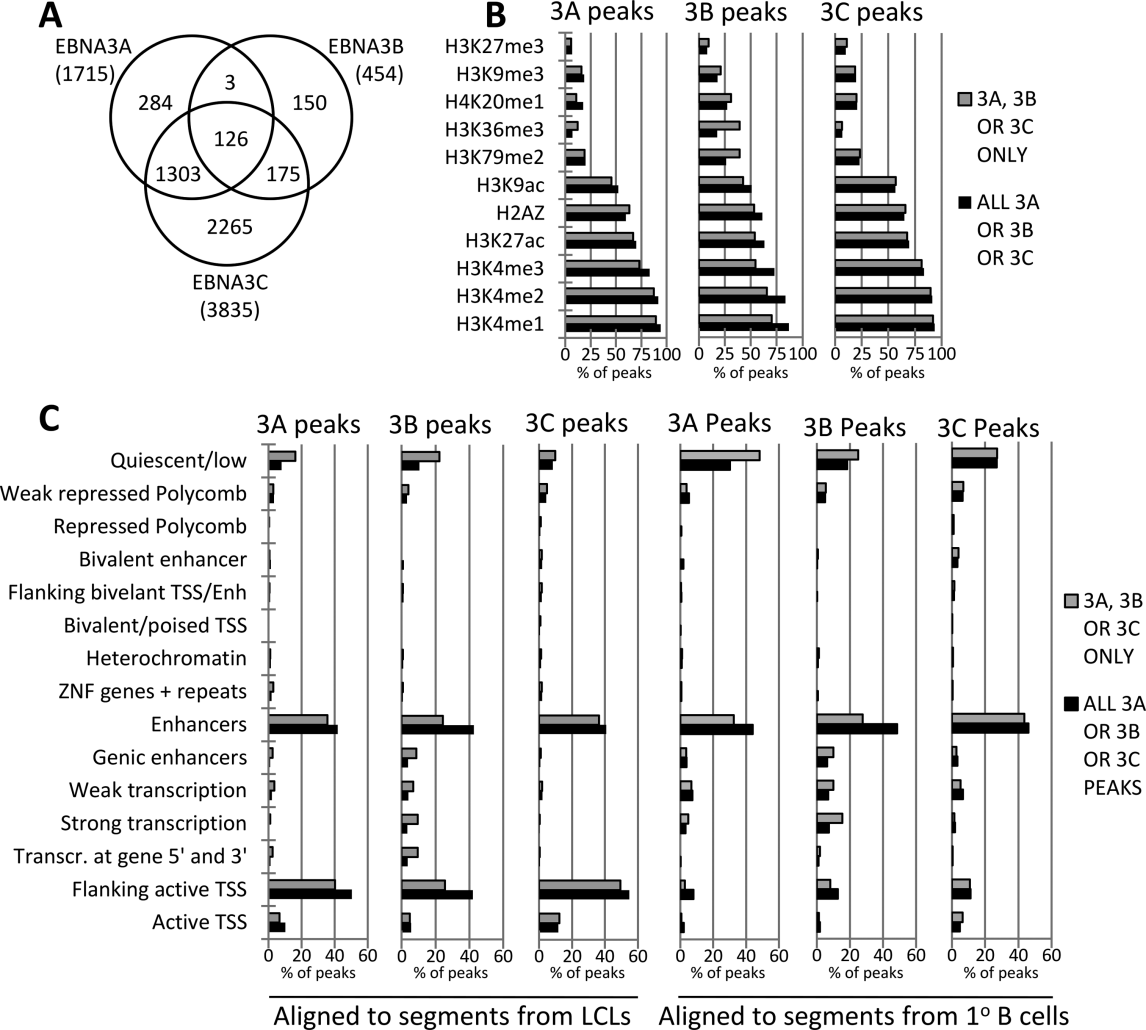
Co-localization of EBNA3A, EBNA3B and EBNA3C peaks and their association with enhancer elements and regions flanking active TSS. (**A**) Total numbers of peaks for each EBNA3, as determined by the MACS algorithm, and the degree to which they co-localize. Peaks were classed as co-localized if they had one or more bp in common. (**B**) Co-localization with histone modification marks of LCL GM12878 from the ENCODE project. Total peaks for each EBNA3 and regions with only one EBNA3 (EBNA3A-, EBNA3B- or EBNA3C-only) considered separately. The length of each histogram bar represents the percentage of peaks co-localizing with each histone modification. Many peaks co-localize with more than one histone modification mark because many regions include more than one modification. (**C**) Co-localization of EBNA3 peaks with genome segments corresponding to 15 different chromatin states that have been characterized as part of the ENCODE project. Peaks found by ChIP-seq in LCL where aligned to chromatin states in LCL and CD19+ve 1° B cells. Total peaks and ‘only’ peaks for each EBNA3 were considered separately.

For the ChIP, a commercial antibody directed against the FLAG epitope was used. As a negative control, the same ChIP procedure was performed on an LCL of the same genetic background (i.e. same donor) infected with EBV that does not express tagged proteins. Pooled ChIP DNA from eight (3C-TAP, TAP-3B) or 12 (3A-TAP and non-tagged) precipitation experiments was then tested by QPCR to verify that there was significant enrichment at known targets of EBNA3A, EBNA3B and EBNA3C that could be detected in the material from tagged cell lines relative to the non-tagged LCL (Figure 1A). The ChIP DNA was then subjected to next generation sequencing and reads produced were aligned to version hg19 of the human genome (Figure 1B for examples). EBNA3 peaks were called for each of the three proteins by using the reads obtained from the non-tagged LCL as background control (Figure 1C and Supplementary Figure S2 for examples). After alignment of the sequenced reads, the MACS algorithm was used to identify peaks. Specifically for EBNA3B using the algorithm was used with the broad peak setting because there were 64 peaks called with the broad peak setting, that were not recognized without this setting (49). The same trends were not observed for EBNA3A and EBNA3C. However, using the broad peak setting, a number of peaks that were close together were recognized as a single peak and these were then manually delineated (23 peaks—Supplementary File S1). Applying the MACS algorithm, 1715 peaks were identified for EBNA3A, 454 for EBNA3B and 3835 for EBNA3C (Figure 2A). There is a significant co-localization between EBNA3A and EBNA3C (1429 peaks co-localized) and between EBNA3B and EBNA3C (301 peaks co-localized). However, EBNA3A and EBNA3B were almost never found together without EBNA3C also being present (only three peaks co-localized—Figure 2A). This indicates a different mode of recruitment for EBNA3A and EBNA3B or inability of one to recruit the other in the absence of EBNA3C.

Extensive ChIP-seq data are available from the ENCODE project for an LCL, GM12878 [https://www.encodeproject.org/ (30)], including data for several histone modifications. These data (Supplementary File S2) were used to determine the association of each EBNA3 with regions rich in specific histone modifications. We, like others (27–29), have found that all three EBNA3s largely co-localize with histone modifications associated with active enhancers—identified by H3K27ac and H3K4me1 and with promoters of actively transcribed genes—identified by H3K4me2, H3K4me3, H3K27ac, H3K9ac and histone variant H2A.Z (50) (Figure 2B). When we compared, here for the first time, all EBNA3 peaks with peaks where only a single EBNA3 was present, for EBNA3A and EBNA3C we saw that the trends for histone mark association were very similar (Figure 2B). However, EBNA3B-only peaks have a broader distribution, including histone marks associated with gene bodies, such as H4K20me1 (51), H3K36me3 and H3K79me2 (52) (Figure 2B). The most remarkable difference between EBNA3B-only and total EBNA3B peaks was the greater association of EBNA3B-only peaks with H3K36me3, a histone mark linked with exonic regions (52,53) (Figure 2B).

We next took advantage of the data from the Roadmap Epigenomics Consortium (54) (Supplementary File S2). These include the segmentation of the genome for different cell types, according to chromatin state, as determined by different histone marks, DNA methylation and DNA accessibility. A 15-state model was created by the Consortium,assigning genomic regions to one of eight active or seven repressed states. The cell types included LCL GM12878 and CD19-positive primary B cells from peripheral blood—the cell type we used to create LCLs by infection with EBV. When the peaks for each EBNA3 were aligned to the chromatin states for an LCL, unsurprisingly the great majority were found at regions classed as enhancers and at—or near—active transcription start sites (TSS), with a minority at regions of quiescent chromatin (Figure 2C). EBNA3B-only peaks have a distinctly different distribution, with markedly less at enhancers and TSS than the other categories of peaks and more at quiescent regions and at regions of active transcription. Aligning the same peaks to chromatin states from primary B cells revealed that many regions of EBNA3 peaks in LCLs classed as active TSS, were classed as quiescent in primary B cells (Figure 2C). This suggests that many quiescent TSS in non-infected primary B cells become active following infection and targeting by the EBNA3s.

### Global chromatin looping data reveals direct and indirect regulation by EBNA3s

**Figure 3.**
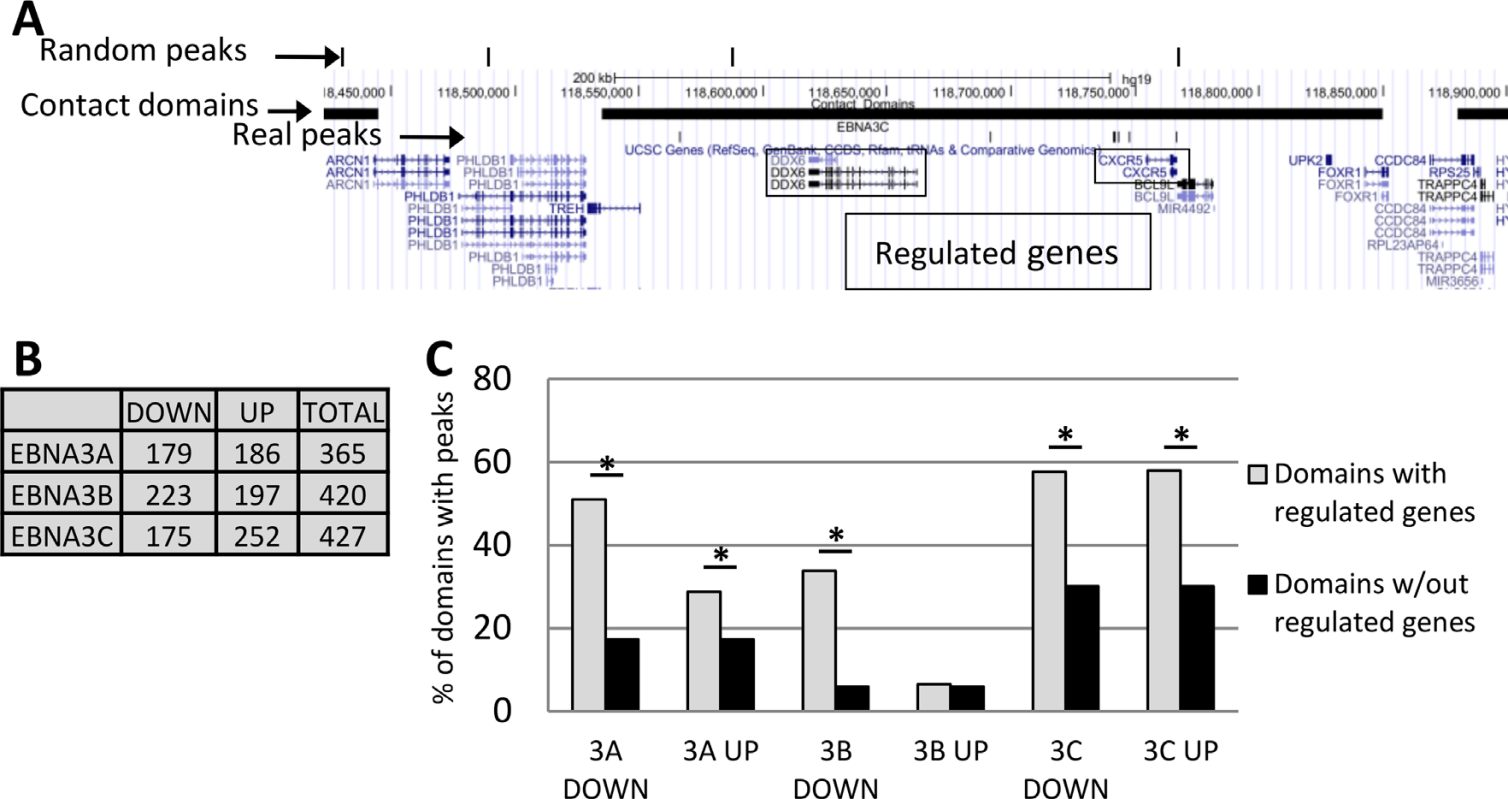
Assessment of direct and indirect regulation by EBNA3s by combining microarray, ChIP-seq and Hi-C data. (**A**) Schematic from the UCSC genome browser with the EBNA3C peaks, random peaks and contact domains tracks for illustration. The contact domain at the centre contains two EBNA3C down-regulated genes—DDX6 and CXCR5. It also contains other genes that show no evidence of EBNA3 regulation. (**B**) Numbers of the most robustly EBNA3-regulated genes, as determined by previous microarray transcriptome studies, divided into down- (repressed) and up-regulated (activated) genes. (**C**) In light gray histogram bars, the percentages of contact domains containing regulated genes also containing the relevant EBNA3 peaks are shown. The black bars represent the percentages of contact domains not containing the relevant EBNA3-regulated genes (up- or down-regulated), but containing EBNA3 peaks. Pearson's chi-squared test was performed to assess the difference in co-localization of peaks with contact domains, either containing or not containing relevant regulated genes. (*) denotes *P* < 0.05 of difference occurring by chance.

To relate the peaks identified by our ChIP-seq with EBNA3-regulated genes revealed by microarrays from LCLs (10,15,17) data from a study revealing global chromatin looping in an LCL (31) were used. The outputs of this study included the partitioning of the LCL genome into contact domains—regions with significant long-range associations within them, suggesting chromatin looping. The median length of the contact domains was 185 kb (see Figure 3A for example). We identified contact domains that contain known EBNA3-regulated genes and ChIP-seq peaks thus linking genes with peaks that are not necessarily close, but for which there is evidence of association by chromatin looping. EBNA3 up-regulated and down-regulated genes were considered separately, 365 genes for EBNA3A, 420 genes for EBNA3B and 427 genes for EBNA3C (Figure 3B). These genes were all found in contact domains and were differentially expressed by at least 2-fold and/or with *P* ≤ 0.001, between LCLs expressing or not expressing the relevant active EBNA3 (10,15,17; http://www.epstein-barrvirus.org.uk/).

The percentage of contact domains that contain EBNA3 peaks and also the relevant regulated genes was calculated (Figure 3C). This was found to be significantly higher than the percentage of contact domains that contain EBNA3 peaks, but do not contain up- or down-regulated genes for each EBNA3, in all cases apart from the case of EBNA3B-up-regulated genes (Figure 3C). This implies that there is a specific enrichment of peaks at domains with regulated genes, apart from domains with EBNA3B-activated genes.

To further assess the significance in the associations detected, random ‘peaks’ were generated for each EBNA3. These were a list of genomic locations—the same in number as the real peaks, with the same lengths as the real peaks, but with their locations determined randomly. The percentage of contact domains with EBNA3-regulated genes that also contain real peaks is considerably higher than for the same domains also containing randomly permuted ‘peaks’, in every case, apart from domains containing EBNA3B up-regulated genes (Supplementary Figure S3). Therefore, there is a positive enrichment for real peaks associated with EBNA3-regulated genes, apart from EBNA3B up-regulated genes. The random ‘peaks’ established a baseline: the number of regulated gene-containing domains found by chance by the EBNA3 peaks. This was in all cases remarkably similar to the percentages of domains without regulated genes that associated with real peaks (compare Figure 3C to Supplementary Figure S3). That is, real peaks behave like random peaks for domains without EBNA3-regulated genes, indicating that the peaks we identified do not regulate a significant number of additional genes we are not considering. Surprisingly, EBNA3B is probably activating genes indirectly, since no significant direct association between EBNA3B peaks and EBNA3B up-regulated genes could be found.

### Contact domains that include EBNA3-regulated genes are either activated or repressed, but not both

There are contact domains that contain more than one EBNA3-regulated gene. For each EBNA3, all such domains were identified, as was the direction of regulation for each EBNA3-regulated gene within them (Table 1). Remarkably, for every one of those domains, all the EBNA3-regulated genes within a single domain were regulated in the same direction (activated or repressed). The only exception is one domain that contains EBNA3B up- and down-regulated genes—but since we determined that EBNA3B is probably only a repressor, the up-regulated genes within that single domain are likely to be regulated indirectly. This conclusion is further supported by the observation that there are no other contact domains containing more than one EBNA3B up-regulated gene, whereas there are 14 contact domains that contain multiple EBNA3B down-regulated genes (Table 1). For both EBNA3A and EBNA3C there are very similar numbers of contact domains containing either activated or repressed genes (Table 1).

**Table 1.** Contact domains containing more than one EBNA3-regulated gene

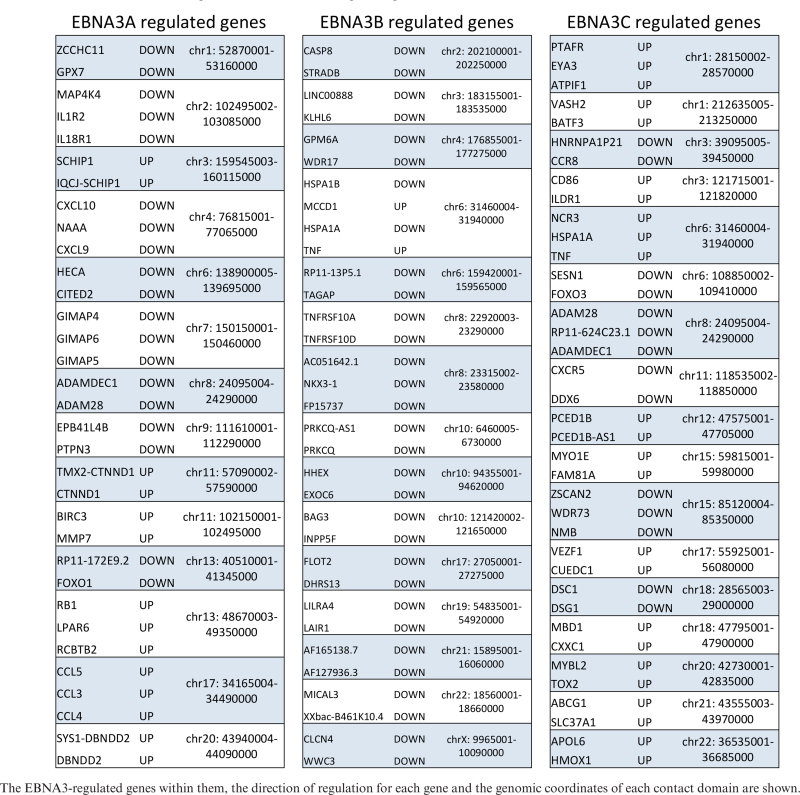

### EBNA3s co-localize with similar transcription factors

**Figure 4.**
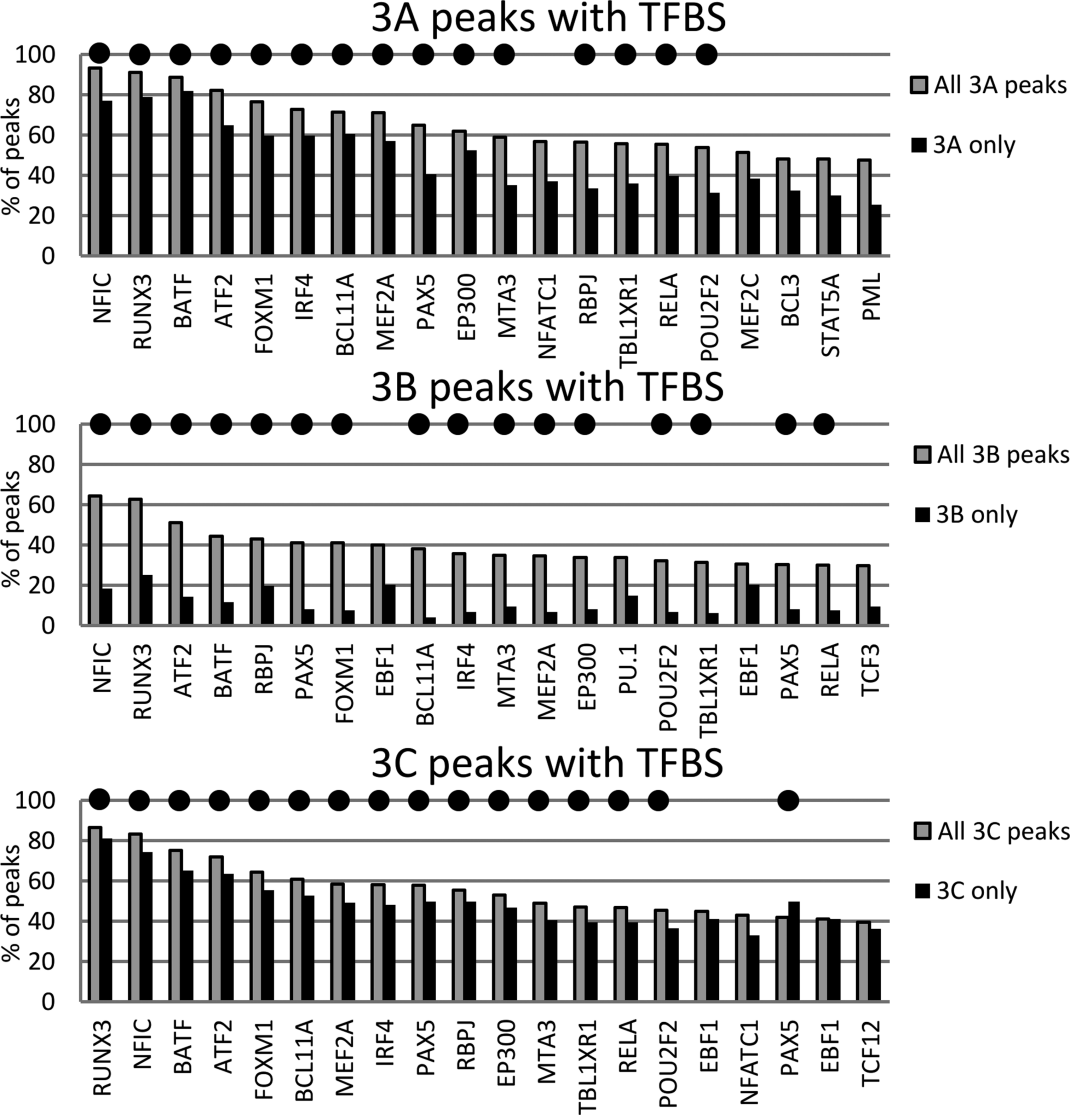
Co-localization of EBNA3 peaks with transcription factors reveals striking similarities between EBNA3s. Total numbers of peaks for each EBNA3 and regions with only one EBNA3 (EBNA3A-, EBNA3B- or EBNA3C-only) were considered separately. The height of histogram bars represents the percentage of peaks that co-localize with each transcription factor binding site (TFBS). The 20 factors most commonly co-localized with the total number of binding peaks for each EBNA3 are shown. Black dots above bars indicate factors that are in the top 20 for all 3 EBNA3s. For transcription factors appearing more than once, more than one track from independent experiments were available from ENCODE.

Extensive ChIP-seq data are available for the localization of cell transcription factors in LCL GM12878. Data from 90 different ChIP-seq experiments for transcription factor localization, uniformly analysed and available from the ENCODE project (29, Supplementary File S1) were used to assess co-localization with each of the EBNA3s. ChIP-seq data for transcription factor RBPJ from a separate study (46) was also used (this was performed using the LCL IB4 that expresses no EBNA3B). The total number of peaks for each EBNA3 was considered and the 20 transcription factors most frequently co-localizing with each are shown (Figure 4). EBNA3A-only, EBNA3B-only and EBNA3C-only peaks were also compared to the same transcription factor binding sites (Figure 4). Co-localization with cell transcription factors is remarkably similar between the EBNA3s, with the majority of the top 20 factors common to all three (Figure 4). When considering co-localizations of EBNA3A or EBNA3C peaks with transcription factors in domains with up-regulated or down-regulated genes separately, the same frequencies are retained (not shown) and therefore, these transcription factors could not be specifically correlated with either activation or repression. Considering EBNA3B-only peaks separately, it might be significant that the factor seventh most frequently co-localized is RNA polymerase II [(POLR2A), Supplementary Figure S4)], which is not in the top 20 of the other EBNA3s, reflecting the association of EBNA3B-only peaks with transcribed regions. It should also be noted that co-localization of EBNA3B-only peaks with the transcription factors considered is low, with the top factor only co-localizing with 25% of EBNA3B-only peaks (Figure 4 and Supplementary Figure S4). However, the most frequently co-localized factor with EBNA3B-only peaks is still RUNX3, which is also most frequently co-localized with EBNA3A-only and all EBNA3C peaks (Figure 4).

### EBNA3B and EBNA3C each co-immunoprecipitate with CBFβ, the partner of RUNX3 in Core Binding Factor (CBF)

**Figure 5.**
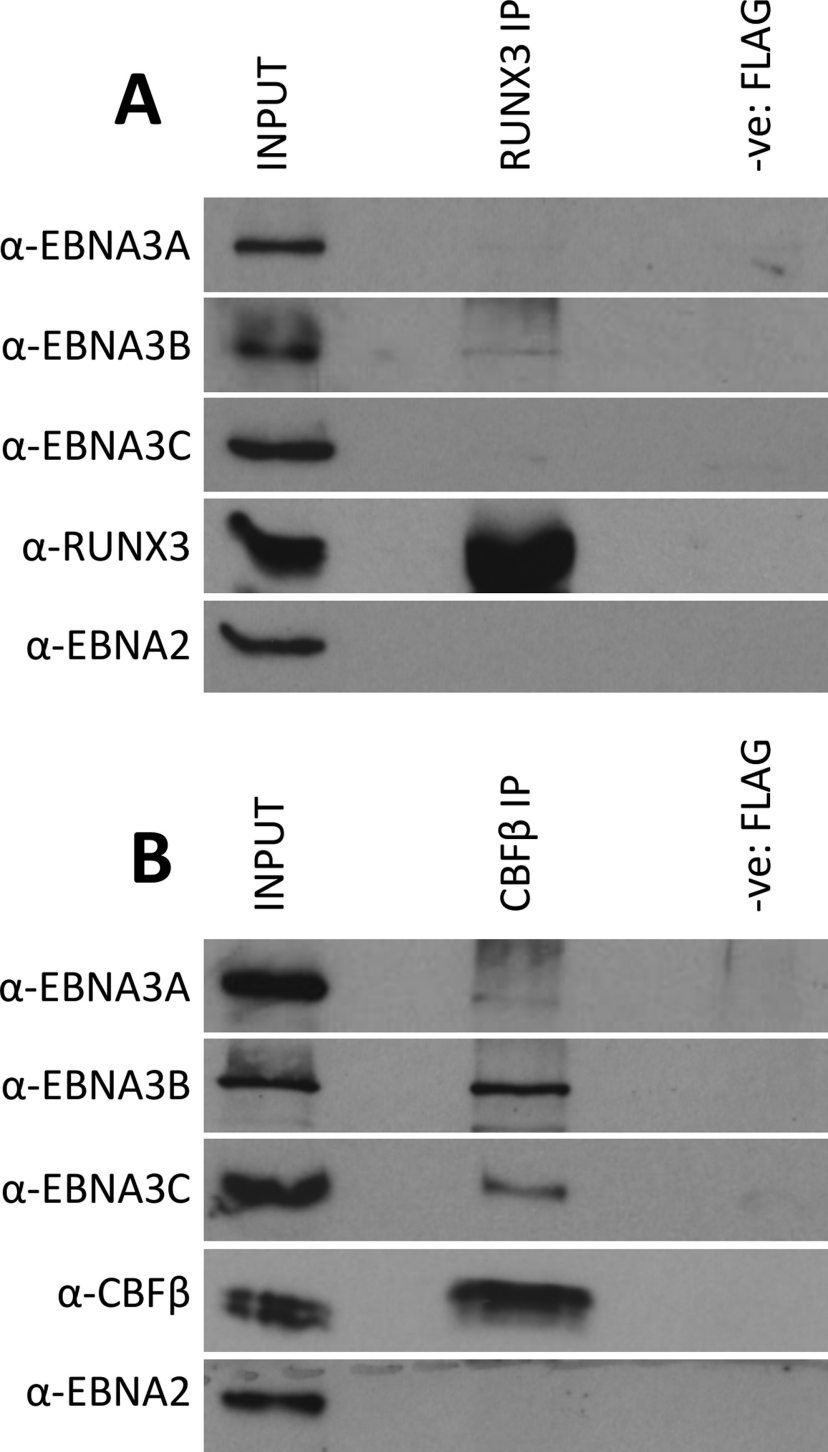
EBNA3B and EBNA3C co-immunoprecipitate with CBFβ in WT LCL. (**A**) RUNX3 was immunoprecipitated using a rabbit anti-RUNX3 antibody and the precipitate was separated by SDS-PAGE and probed by western blotting for EBNA3A, EBNA3B, EBNA3C or, as a positive control, RUNX3, using non-rabbit antibodies; 10% of input sample is shown for comparison. A rabbit anti-FLAG antibody was used for the same non-tagged cell line lysate as negative control (-ve: FLAG). (**B**) CBFβ was immunoprecipitated using a rabbit anti-CBFβ antibody. The precipitate was separated by SDS-PAGE and probed by western blotting for EBNA3A, EBNA3B, EBNA3C, CBFβ or EBNA2; 10% of the input sample is shown for comparison and for negative control an anti-FLAG immunoprecipitation was used as in (A).

Expression of RUNX3 is induced during the infection of B cells, because the gene encoding this transcription factor is a direct target of the EBV transactivator EBNA2 (55–57). With this in mind—and because RUNX3 extensively co-localizes with EBNA3s on chromatin across the human genome—we wanted to explore whether any of the EBNA3s can be found in complexes with RUNX3. An anti-RUNX3 antibody was used to immunoprecipitate RUNX3 from extracts from LCLs infected with wild type EBV. However, after repeated attempts no EBNA3s were reproducibly or convincingly co-immunoprecipitated with RUNX3 (Figure 5A).

As mentioned before, RUNX proteins bind to DNA as a heterodimer with CBFβ and together they form core-binding factor (CBF). Consequently, an anti-CBFβ antibody was used to immunoprecipitate CBFβ from LCL protein extracts and, as expected, RUNX3 co-immunoprecipitated with CBFβ (not shown). EBNA3A co-immunoprecipitated very weakly with CBFβ, but EBNA3C and EBNA3B did so significantly—the latter most robustly (Figure 5B). EBNA2, although it can sometimes co-localize with the EBNA3s on chromatin (27,29), did not co-immunoprecipitate with CBFβ under the same conditions (Figure 5B). Similar immunoprecipitation experiments were performed in LCLs infected with a recombinant EBNA3C- or EBNA3B-knock-out virus (10,15)) and showed that EBNA3B and EBNA3C can be co-immunoprecipitated with CBFβ independently of each other and that EBNA3A co-immunoprecipitates in the absence of its main co-localizing EBNA3, EBNA3C (Supplementary Figure S5).

### Depletion of RUNX3 or CBFβ reduces recruitment of EBNA3s to EBNA3-regulated gene loci

**Figure 6.**
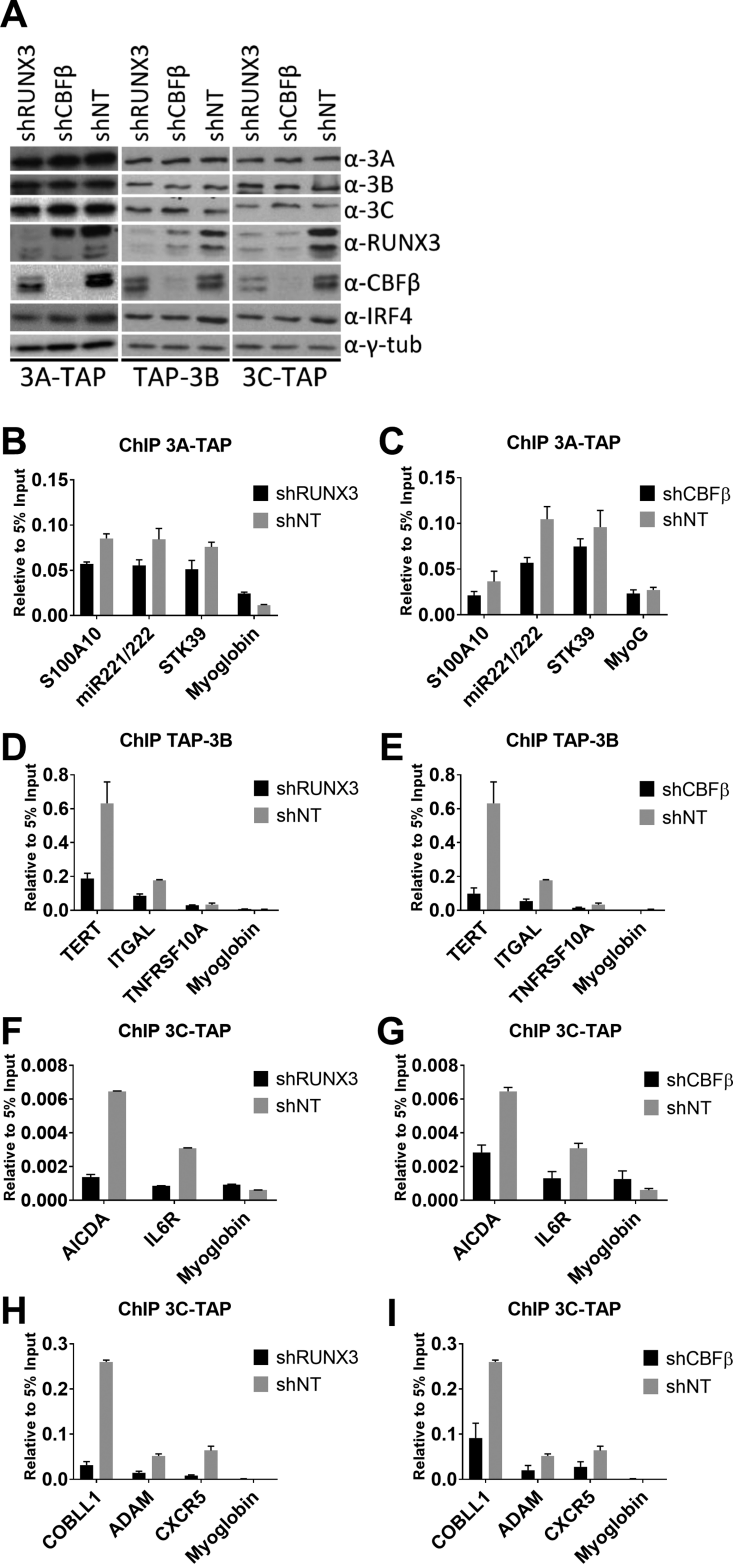
Depletion of RUNX3 and CBFβ disrupts recruitment of EBNA3A, EBNA3B and EBNA3C in the regions of robustly regulated genes. (**A**) EBNA3A-TAP, TAP-EBNA3B-and EBNA3C-TAP tagged expressing LCLs were infected with lentiviruses producing shRNAs that targeted RUNX3 (shRUNX3), CBFβ (shCBFβ) as indicated or producing a control, non-targeting shRNA (shNT) and cells were harvested 6 days after infection with lentivirus. Depletion of RUNX3 and CBFβ was confirmed, and the expression of each EBNA3 and also IRF4 were demonstrated by western blotting. (**B-I**) ChIP analyses using an anti-FLAG antibody in 3A-TAP, TAP-3B and 3C-TAP cell lines with knock-downs as indicated. Histogram bar heights represent fold-enrichment relative to 5% input sample and standard deviations are calculated from QPCR triplicates for each sample. RUNX3 and CBFβ knock-downs have been performed independently with two different shRNAs each, with similar results. Representative examples from the best knock-downs are shown. Primers amplifying a region of the Myoglobin promoter were used as negative control.

Since the EBNA3s all appear in complexes with CBF, lentiviruses that express shRNAs targeting RUNX3 and CBFβ were constructed to assess the role of these factors in EBNA3 recruitment to chromatin. Two independent lentiviruses expressing different shRNAs were produced for each mRNA and these depleted the target proteins to a similar extent in three independent experiments, however here only one of each is shown (Figure 6A). All the lentiviruses also expressed the gene for puromycin resistance. RUNX3 and CBFβ expression was knocked down in the LCLs that express the tagged versions of EBNA3A, EBNA3B or EBNA3C. Puromycin was added 2 days after lentiviral infection and the cells were harvested after a further 4 days. Depletion of both factors was efficient, when compared to the same lines infected with lentiviruses expressing a non-targeting shRNA (Figure 6A). The levels of each tagged EBNA3 were unaffected by the knock-downs, but in the cells with CBFβ depleted, the level of RUNX3 protein was also reduced (Figure 6A). This was consistent with reports that CBFβ stabilizes RUNX proteins (58). The level of IRF4 in the cells did not change with RUNX3 and CBFβ depletion (Figure 6A). IRF4 was tested because it was previously shown to play a role in EBNA3C targeting (24,29,59).

Anti-FLAG ChIPs were performed on these cells, followed by Q-PCR to assess the levels of EBNA3-TAP bound at EBNA3 peaks associated with selected EBNA3-regulated genes (Figure 6B–H). Enrichment was determined relative to input for cells that had RUNX3 or CBFβ knocked down or cells infected with the non-targeting control lentivirus. Reduced levels of each EBNA3 were seen at loci associated with EBNA3-regulated genes (Figure 6B–H), indicating that CBF is important for EBNA3 recruitment to chromatin.

A lentivirus expressing shRNA for IRF4 was also produced, in order to assess the role of this factor in a similar experiment. IRF4 was only knocked down in the 3C-TAP cell line (Supplementary Figure S6A), because it was found previously to direct only EBNA3C recruitment (29). Reduced binding of 3C-TAP around EBNA3C-regulated genes was seen after IRF4 depletion (Supplementary Figure S6B), confirming the role for IRF4 in recruitment, as has been previously suggested (24,29).

As a control for RUNX3, CBFβ and IRF4 depletion experiments, EBNA2 ChIPs were performed in a similar manner, on the same batch of chromatin, after their infection with each shRNA-expressing lentivirus to show that the trend of reduced EBNA3 enrichment after knock-down was specific. EBNA2 enrichment at known EBNA2 peaks was not diminished after knockdown of any of these transcription factors and in most cases more EBNA2 was observed (Supplementary Figure S6C), most likely due to the antagonistic relationship of EBNA3 and EBNA2 recruitment shown previously (60–63).

For EBNA3B, a site where no RUNX3 binds was used as a control to show that diminished binding was only found at CBF sites, after depletion of either RUNX3 or CBFβ (Supplementary Figure S6D). We were unable to find—by ChIP-QPCR—EBNA3A or EBNA3C peaks where CBF binding was not above background (data not shown).

### EBNA3C stabilizes CBF on chromatin

Although we found that efficient localization of EBNA3B, EBNA3C and to a lesser extent EBNA3A is dependent on the presence of CBF, it was unknown how the EBNA3s affect the occupancy of CBF at the same loci. In order to assess this, we used two LCLs expressing either a conditional EBNA3C or a conditional EBNA3A. The use of conditional EBNA3s, allowed us to detect changes soon after expression of active EBNA3s, avoiding the problems of a long LCL outgrowth process that is subject to selection pressures. The conditional EBNA3C LCL is *p16^INK4A^*-null and expresses EBNA3C fused to a modified estrogen receptor (3CHT) that renders 3CHT active only in the presence of 4-hydroxytamoxifen (HT) (10). In the absence of HT, 3CHT is sequestered to the cytoplasm, where it is degraded. This LCL was established after infection with EBV without ever being treated with HT—i.e. without ever expressing functional EBNA3C (3CHT never HT). This is possible because the cells lack *p16^INK4A^*, which would otherwise be induced and arrest cells not expressing EBNA3C (10). The conditional EBNA3A LCL expresses EBNA3A fused to a newer version of the estrogen receptor than the one used for EBNA3C, termed ERT2 (3AERT2) (22,64), and it was also grown out without ever being treated with HT—without ever expressing functional 3AERT2. Unfortunately, attempts to produce a similar conditional EBNA3B-expressing recombinant virus were unsuccessful because of technical problems associated with extending the EBNA3B open reading frame.

**Figure 7.**
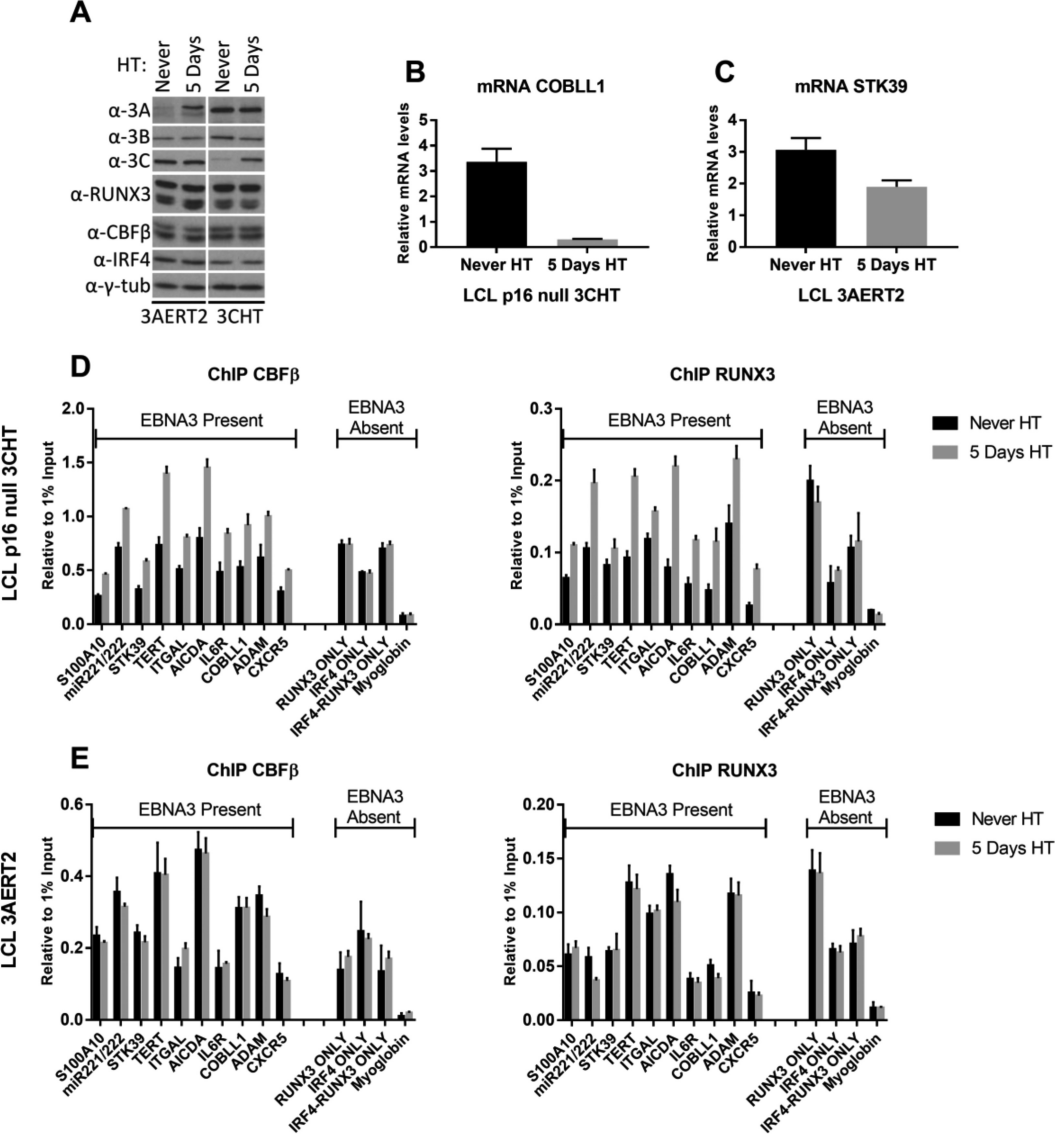
EBNA3C but not EBNA3A stabilizes CBFβ and RUNX3 on chromatin. LCLs carrying either a conditional EBNA3A (3AERT2) or a conditional EBNA3C (3CHT) that are activated by addition of HT to the culture medium were used. Both cell lines were produced with no HT present in the medium from when 1° B cells were infected (Never). HT was added to half the culture to activate either EBNA3A or EBNA3C and cells were harvested after 5 days. (**A**) Activation and stabilization of 3AERT2 and 3CHT proteins was shown by western blotting. No change in levels of EBNA3B, CBFβ, RUNX3 and IRF4 after activation was detected. (**B**) RT-QPCR for mRNA levels of robustly EBNA3C-regulated gene COBLL1 after 5-day activation of 3CHT with addition of HT. (**C**) RT-QPCR for mRNA levels of EBNA3A-regulated gene STK39, following 5-day activation of 3AERT2 with addition of HT. (**D**) ChIP for CBFβ and RUNX3 carried out for Never HT and 5 days HT 3CHT cultures. Enrichment at several EBNA3-regulated genes was assessed. Enrichment at control loci where there was no apparent EBNA3 binding was also assessed. A locus at the promoter region of the Myoglobin gene acts as a control for no enrichment for any of these factors. (**E**) As in (D), but for 3AERT2. For all QPCR, error bars represent standard deviations calculated from triplicates for each sample.

Cultures of 3CHT or 3AERT2 LCL never exposed to HT were split and to one half of each HT was added for 5 days. Cells with and without HT were harvested for protein and RNA extraction or fixed for ChIP. The time course for 3CHT was repeated three times and 3AERT2 twice. Stabilization of 3CHT and 3AERT2 was verified by Western blot (Figure 7A). The ability in these LCLs to regulate mRNA abundance of known EBNA3A or EBNA3C target genes was confirmed by RT-QPCR (Figure 7B and C), demonstrating the functionality of 3CHT and 3AERT2 during the 5 days of the time course. ChIPs for CBFβ and RUNX3 were performed and the enrichment of both was assessed by QPCR (Figure 7D and E). The loci tested contained peaks of RUNX3 (as determined by data available from ENCODE) and EBNA3, or—as controls—loci where there is no evidence of EBNA3 binding (Figure 7D and E). These control loci contained RUNX3 and/or IRF4, or, in the case of myoglobin, did not contain peaks for any of the factors studied. As an additional control, ChIP for EBNA2 was performed at the same time, to show that the trends observed for the factors studied were specific (Supplementary Figure S7).

When HT was added to the LCL 3CHT cells never exposed to HT, occupancy of both CBFβ and RUNX3 was increased at loci where EBNA3 was found, but not at loci with no EBNA3 binding (Figure 7D). Activation of 3AERT2 by the addition of HT did not have this effect—no appreciable increase in either CBFβ or RUNX3 occupancy being observed at any of the chosen target loci (Figure 7E). This indicates that EBNA3C, but not EBNA3A, stabilizes CBF at loci of EBNA3 binding.

### CBF is required for efficient regulation of target genes by EBNA3C

**Figure 8.**
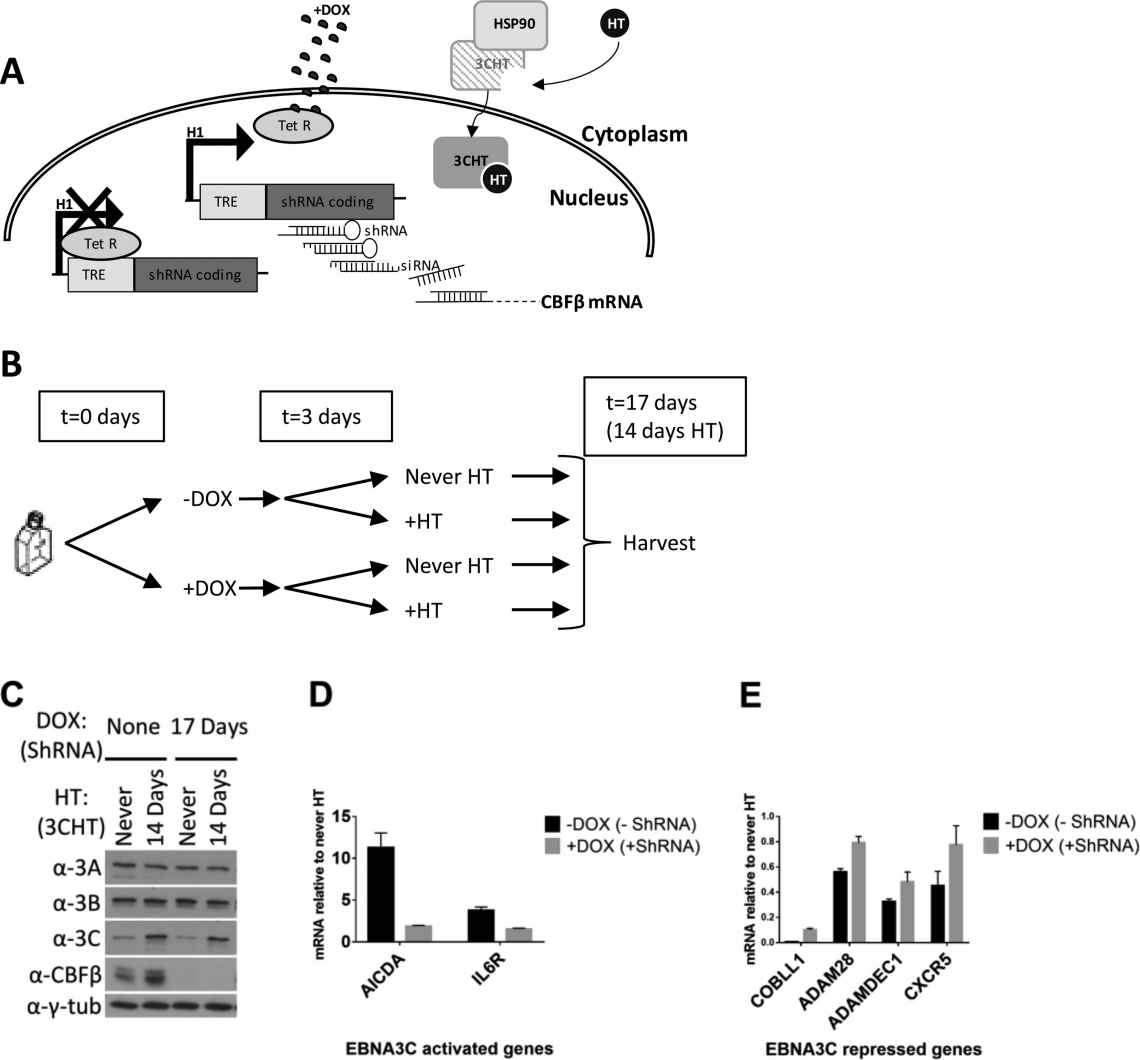
CBF is required for efficient gene regulation by EBNA3C. (**A**) The 3CHT LCL never exposed to HT was used, which expresses a conditional EBNA3C (3CHT—active only after addition of HT). Inducible shRNA targeting mRNA of the CBF subunit CBFβ was used (pLKO-Tet-On). This was generated from a lentiviral construct that constitutively expresses the tetracyclin repressor (TetR). The shRNA can be expressed from an H1 promoter upstream of two Tet Response Elements (TRE). In the absence of the tetracycline analogue doxycycline (DOX), transcription is prevented allosterically by binding of TetR to TRE. Addition of DOX changes the conformation of TetR, which can no longer bind TRE and transcription of the shRNA is possible. After processing, the shRNA mediates knock-down of CBFβ which is no longer available to 3CHT, activated after addition of HT. (**B**) Schematic of the time course experiment: DOX was added to half the culture of the 3CHT LCL (never HT) carrying pLKO-Tet-On to induce expression of shRNA against CBFβ, resulting in two cultures as shown. After 3 days HT was added to half of each culture to activate 3CHT, resulting in the four cultures shown. After 14 days of HT, cells were harvested. (**C**) Western blots of extracts from all 4 resultant cultures, showing efficient knock-down of CBFβ after addition of DOX, stabilization of 3CHT after addition of HT and levels of EBNA3A and EBNA3B proteins. Gamma-tubulin was used as a loading control. (**D**) RT-QPCR of extracted RNA; mRNA levels of EBNA3C up-regulated genes (AICDA and IL6R) after activation of 3CHT, relative to their levels before activation of 3CHT are shown. The mRNA for each gene shown were first normalized to mRNA of endogenous control gene ALAS1, the levels of which do not significantly change throughout the time course (data not shown). (**E**) As in (D), but for genes known to be repressed by EBNA3C (COBLL1, ADAM28, ADAMDEC1 and CXCR5). For all QPCR, error bars represent standard deviations calculated from triplicates for each sample.

EBNA3C was shown to co-immunoprecipitate with CBFβ from whole cell extracts, CBFβ was found to be required for efficient EBNA3C localization to chromatin and EBNA3C was shown to stabilize CBF on chromatin. We therefore wanted to determine whether CBF was also important for EBNA3C to act as a regulator of host genes. For this the pLKO-Tet-On lentiviral system for doxycycline (DOX) inducible knock-down of CBFβ was used (65) (Figure 8A). Two independent lentiviruses were produced with different shRNAs targeting CBFβ (Supplementary Table S1) and the experiment was performed twice for each lentivirus, showing similar results. DOX was added to 3CHT LCL never exposed to HT cells that were also infected with this lentivirus, in order to knock down CBFβ. After 3 days, HT was also added to activate 3CHT (Figure 8B). Fourteen days after activation of 3CHT (by addition of HT)—and 17 days after knocking down CBFβ (by the addition of DOX), cells were harvested for protein and RNA (Figure 8B). Addition of DOX induced a robust reduction in CBFβ protein levels, assessed by western blot, irrespective of the presence of active 3CHT (Figure 8C). Similarly, 3CHT stabilization was assessed, as was the stability of EBNA3A and EBNA3B throughout the time course (Figure 8C). The levels of mRNA were determined by RT-QPCR for several genes known to be either up-regulated (Figure 8D) or down-regulated (Figure 8E) by EBNA3C. Relative to cells that had never expressed active 3CHT, activation of EBNA3C-regulated genes was significantly less pronounced when CBFβ was depleted (Figure 8D). Concurrently, impaired repression of EBNA3C-down-regulated genes was also seen when CBFβ was depleted (Figure 8E). Together these data unequivocally establish that CBF is necessary for the regulation of host genes by EBNA3C.

## DISCUSSION

In this study, we performed ChIP-seq to assess genome-wide localization of EBNA3A, EBNA3B and EBNA3C. Similar studies have revealed many aspects of the binding-site characteristics and of EBNA3 function (24,27–29). Here, an experimental protocol that significantly improves on previous studies was used. We were able to ChIP each of the three EBNA3s, using the same antibody—since they were all tagged with the same tandem affinity purification tag—in the same genetic background and with cells proliferating equally well in all instances. This is the first time this has been achieved. Additionally, ChIP DNA from a LCL infected with wild type EBV expressing non-tagged proteins provided a more refined control for DNA accessibility and sequencing bias as well as for non-specific antibody binding. Finally, for peak calling we used the MACS algorithm (45), which is also used by ENCODE (30) in their uniform peak calling pipeline.

Comparing the EBNA3 MACS peaks called, we see that in general there is significant co-localization between EBNA3C and EBNA3A (83% of -3A co-localized) and between EBNA3C and EBNA3B (66% of -3B co-localized), but it appears that any co-localization between EBNA3A and EBNA3B occurs in the presence of EBNA3C, with only three regions of EBNA3A-EBNA3B exclusive co-localization (Figure 2). The epigenetic landscape at regions of EBNA3 peaks and the chromatin state derived from this landscape are very similar between EBNA3s, with a strong association with enhancer and TSS regions (Figure 2B and C). However, the decision to consider separately peaks with only one EBNA3 present revealed that EBNA3B-only peaks are qualitatively different, showing preference for chromatin characteristic of gene bodies and exonic regions in particular (Figure 2B and C). The significance of this has not yet been determined, but the majority (87%; data not shown) of EBNA3B repressed genes do not have EBNA3B peaks within their bodies, suggesting that the peaks within gene bodies are distally targeted and subject to chromatin looping, rather than directly repressing the genes in which they are found.

Another striking observation concerning EBNA3 peaks was revealed by examining the same loci, but in uninfected primary B cells (Figure 2C). For all EBNA3s, a significant proportion of peak regions lie close to TSS determined to be quiescent in primary B cells. However, there is no evidence that a significant number of these TSS belong to EBNA3 activated genes, with no more than 10% (data not shown) for any EBNA3. The change in chromatin character probably facilitates EBNA3 targeting, but it does not necessarily seem to be caused by the EBNA3s, because in that case these genes would have been identified as EBNA3-activated genes in the microarray studies. Therefore, this change in chromatin character might be brought about by the process of B cell activation itself, facilitating EBNA3 binding to distal sites—the EBNA3s then exerting their influence following chromatin looping to other genes and/or regulatory elements.

Chromatin looping seems to be a very important mediator of EBNA3 function, as has been shown previously for individual genes (22,27). It has been assumed that this effect is genome-wide because the EBNA3s associate very frequently with enhancer regions (24,27–29). To explore the role of chromatin looping in EBNA3 function globally, we used the chromatin contact domains discovered for LCL GM12878 recently (31), in order to relate EBNA3 peaks to known, robustly regulated genes by each EBNA3 (see results). Although this sort of analysis could be obfuscated by either too stringent or too lax cut-off criteria or technical limitations, in this case the statistical significance of peak enrichment in contact domains with EBNA3-regulated genes was obvious and strong (Figure 3C). There are significantly more peaks in contact domains with regulated genes than in domains without the regulated genes we considered. This was true for all categories of genes, apart from EBNA3B-activated genes, indicating that EBNA3B might not up-regulate genes by direct contact with their genomic location. There are two, non-mutually exclusive, possibilities for EBNA3B-mediated activation without direct contact with activated genes. Either EBNA3B activation of genes results from EBNA3B directly repressing other genes, or EBNA3B disrupts chromatin looping that would have caused repression, had EBNA3B not been there. Indeed, prevention of looping by the EBNA3s has been shown directly (27) and an element of this could be present for all classes of EBNA3-regulated genes, especially in the EBNA3A-activated genes, where the enrichment for peaks is significant, but not as pronounced as for others (Figure 3C). The conclusions of this analysis are reinforced by similar trends observed when using random ‘peaks’, although here a similar statistical analysis would not be appropriate. This is because more than one real peaks can be observed within a single domain. This is scored as one positive with real peaks, but could produce more positive hits with random peaks [independence of outcome violated (47)]. More significantly, the conclusion that EBNA3B-mediated activation is indirect is supported by the observations made when considering contact domains with more than one regulated genes, with almost no such EBNA3B up-regulated genes observed (Table 1).

Contact domains with more than one regulated gene revealed another important aspect of EBNA3 regulation, namely that regulated genes in contact with the same peaks are regulated in the same direction, either all being activated or all being repressed. This implies that factors determining the direction of regulation reside at the peak region and are not dependent on the *cis* context of the gene proximal regions, which might only be relevant in determining whether looping occurs, but not for its functional outcome.

ChIP-seq data also highlighted the CBF component RUNX3 as the most commonly co-localising factor with the EBNA3s and an obvious candidate for further study. This seemed particularly germane since infection of B cells with EBV changes the composition of CBF, from RUNX1-CBFβ heterodimers to RUNX3-CBFβ heterodimers, by inducing RUNX3 and suppressing RUNX1 (55). EBNA2 (and probably EBNA3B and EBNA3C) plays a direct role in this effect (55–57) and RUNX3 subsequently mediates the repression of RUNX1 (56,66–69). The composition of CBF appears to be associated with B cell differentiation stages (69–71). The switch from RUNX1 to RUNX3 is important for LCL proliferation, since siRNA depletion of RUNX3 (leading to increase of RUNX1) or exogenous expression of RUNX1 results in impairment of LCL outgrowth (56,67). We sought to test the hypothesis that RUNX3-containing CBF, in addition to repressing growth-restricting RUNX1, is also directly involved in EBNA3 functions.

Co-immunoprecipitation of EBNA3A, EBNA3B and EBNA3C with RUNX3 partner CBFβ supported this hypothesis. There is a stronger physical association with EBNA3B than with EBNA3C (or EBNA3A—which co-precipitates very weakly under the same conditions) (Figure 5). Although we have shown that EBNA3B and EBNA3C can co-immunoprecipitate independently of each other (Supplementary Figure S5), it remains unclear why co-localization studies suggest the reverse order of association with CBF. A possible explanation is that one EBNA3 can recruit one or both of the others to some sites since there is evidence of the EBNA3s physically interacting with each other in solution (42). Alternatively, or in addition, there could be combinations of multiple factors targeting the EBNA3s to regions of the genome. The important observation is that for the first time we also show that depletion of RUNX3 or its co-factor CBFβ (knockdown of which also reduces levels of RUNX3) leads to a reduced recruitment of EBNA3A, EBNA3B and EBNA3C (Figure 6).

However, it also seems that the relationship between EBNA3C and CBF during recruitment to chromatin is reciprocal—EBNA3C also facilitates robust stabilization or increased binding affinity of RUNX3 and CBFβ onto chromatin (Figure 7). This is reminiscent of the relationship between EBNA3C and EBNA2 with RBPJ (26,72), a factor long known to interact with EBNA2 and all EBNA3s (reviewed in 5) and thought to play a role in their recruitment to chromatin—but probably only at a subset of binding sites (29,72). A question of cause and effect arises. None of the EBNA3s appear to have a DNA binding domain (21 and our unpublished data), so they must require DNA binding factors to associate with chromatin specifically and efficiently. A combination of factors is likely to decide EBNA3 recruitment. The ChIP-seq data on RUNX3 and the EBNA3s suggest that there are thousands of RUNX3 binding sites with no evidence of EBNA3 binding, consistent with a requirement for additional factors. There must also be a role for other *cis* elements making regions of the genome more susceptible to EBNA3 binding. These are likely to be where chromatin is open or opens as a consequence of B cell activation, thus being available for binding of multimeric complexes containing EBNA3s and then being stabilized as a result of combinatorial binding.

Irrespective of the precise sequence of events or variety of factors involved, CBF is clearly very important. The high degree of co-localization, the co-immunoprecipitation and the requirement for CBF in efficient targeting of EBNA3B and EBNA3C to chromatin are consistent with this. Moreover, it could be significant that CBF has also been shown to directly recruit polycomb repressive complex 1 (PRC1) subunit BMI1 (73), a factor also shown to affect EBNA3 gene regulation (42). Finally, we show (Figure 8) that CBF is required for both activation and repression of host genes by EBNA3C. These data definitively establish CBF as a central component of the complex gene regulatory network that links EBNA2, the EBNA3 family and multiple host genes during EBV latency and B cell transformation.

## ACCESSION NUMBER

The ChIP-seq data reported in this paper have been deposited in the GEO database under accession number GSE88729.

## Supplementary Material

Supplementary Data
