# Supplementary material for: Core binding factor (CBF) is required for Epstein-Barr virus EBNA3 proteins to regulate target gene expression
Source: Nucleic Acids Res. 2016 Nov 28;45(5):2368–83. doi: 10.1093/nar/gkw1167 (PMC5389572; doi:10.1093/nar/gkw1167)
Supplement: Supplementary Data [file gkw1167_Supp.zip › Suppl_Figures_and_Tables_resubm14-11.pdf]

Supplementary Table S1

Name	orientation	sequence (5'-3')	Results shown
NT	For	CCGGCCTAAGGTTAAGTCGCCCTCGCTCGAGCGAGGGCGACTTAACCTTAGGTTTTTG	YES
NT	Rev	AATTCAAAAACCTAAGGTTAAGTCGCCCTCGCTCGAGCGAGGGCGACTTAACCTTAGG	YES
IRF4	For	CCGGCCGCCATTCCTCTATTCAAGACTCGAGTCTTGAATAGAGGAATGGCGGTTTTTG	YES
IRF4	Rev	AATTCAAAAACCGCCATTCCTCTATTCAAGACTCGAGTCTTGAATAGAGGAATGGCGG	YES
CBFB-1	For	CCGGGAGAAGCAGGCAAGGTATATTCTCGAGAATATACCTTGCCTGCTTCTCTTTTTG	YES
CBFB-1	Rev	AATTCAAAAAGAGAAGCAGGCAAGGTATATTCTCGAGAATATACCTTGCCTGCTTCTC	YES
CBFB-2	For	CCGGTGACCTCAAACTTCGTTAATTCTCGAGAATTAACGAAGTTTGAGGTCATTTTTG	NO
CBFB-2	Rev	AATTCAAAAATGACCTCAAACTTCGTTAATTCTCGAGAATTAACGAAGTTTGAGGTCA	NO
RUNX3-D	For	CCGGACCACCTCTACTACGGGACATCTCGAGATGTCCCGTAGTAGAGGTGGTTTTTTG	YES
RUNX3-D	Rev	AATTCAAAAAACCACCTCTACTACGGGACATCTCGAGATGTCCCGTAGTAGAGGTGGT	YES
RUNX3-E	For	CCGGGTTCAACGACCTTCGCTTCGTCTCGAGACGAAGCGAAGGTCGTTGAACTTTTTG	NO
RUNX3-E	Rev	AATTCAAAAAGTTCAACGACCTTCGCTTCGTCTCGAGACGAAGCGAAGGTCGTTGAAC	NO

Supplementary Table S2

Target	Product Information	Application	Amount/ dilution
EBNA3A	Abcam, ab16126	WB	1:1000
EBNA3B	Clone 6C9, Allday Lab, E. Kremmer {White, 2010 #15}	WB	1:10
EBNA3C	Clone A10, kind gift M. Rowe, University of Birmingham	WB	1:10
γ-tubulin	Sigma, T6557	WB	1:8000
RUNX3	Santa Cruz, sc-101553	WB	1:1000
RUNX3	Abcam, ab11905	IP	2μg
RUNX3	Kind gift Prof Y. Groner, Weizmann Institute of Science	ChIP	4μg
CBFβ	Bethyl, A303-547A	WB/Chip	1:1000/4μg
EBNA2	Abcam, ab90543	WB/Chip	1:1000/4μg
IRF4	Millipore, 04-142	WB	1:1000
FLAG	NEB, 2368	ChIP/IP	4μg/2μg
nornal rab	Millipore, PP64B	ChIP/IP	4μg/2μg
EBNA1	Human serum, kind gift Prof. P Farrell, Imperial College London	WB	1:750
LMP1	Dako, CS1-4	WB	1:500
WB: western blot			
IP: immunoprecipitation			
ChIP: chromatin immunoprecipitation			

Supplementary Table S3

Application	Gene	Forward primer (5'-3')	Reverse primer (5'-3')	Reference
RT-QPCR	IL6R	TTGTTTGTGAGTGGGGTCCT	TGGGACTCCTGGGAATACTG	
	AICDA	TGTCACGCCTCTTCACTACG	TGCAAATAGCCATCATGACC	(1)
	COBLL1	CTGTTCAGCTGACAACAGATCG	ACGTTGAACTCTCAGTGGTCCT	(2)
	ADAM28	GTTGCAGGGACAATGGCACA	TGAGACGGCTGCAGGAACTG	(2)
	ADAMDEC1	CCTTGGTATGCCTGATGTTCCA	CAGCAGGCACTTTGGTTTCTGA	(2)
	CXCR5	AACTACCCGCTAACGCTGGAAATGGAC	CACGGCAAAGGGCAAGATGAAGACC	
	GNB2L1	GCTTGCAGTTAGCCAGGTTC	GAGTGTGGCCTTCTCCTCTG	(3)
	ALAS1	TCCACTGCAGCAGTACACTACCA	ACGGAAGCTGTGTGCCATCT	(3)
ChIP	AICDA	GCTAGTTAACTTTGTTGATC	CTACTCAGGACAGAAATGAC	(1)
	IL6R	GCTCCTGAGTGGATGCTAGG	CCCCAGTCTCTCAGTTCTGC	
	COBLL1	CTGAGTAACAAGAGCGAAAGAG	ATCAGATGTGTTATGACTAACAGC	(2)
	ADAM	CTTCATGGCTACAGACTCTTGG	CCTATGTCTCGCTTCCTGCT	(4)
	CXCR5	GAGGCAGAACAACAGCAACA	AGGTTTGGTGAATGGATGGA	
	S100A10	ACGCAGTGTTTCAAGTGTGC	ACCCACCAAGAGCTGCATAG	
	miR221/222	GAGCAAACTAAGCTCTCCTCCACACA	CGCCTTTGGCTGAAACTGACTG	(5)
	STK39	TCACTTTTACATCCTCGTTGTCAAG	TTTGTTGCCCAGGTTATGTC	
	TERT	CAAAGAGAAGCCACCCAGAG	TCTGGTGGGTGTGTAGTGGA	
	ITGAL	TGCTTACACTTCCTCCCTGAA	TTTCTCACAGAGGCAACAGG	
	TNFRS10A	AAGTCCACACAGGCAGGTTC	TGACCTCACCTTCCATCTCC	
	HES1	AAGTTTCACACGAGCCGTTC	GCTGTTATCAGCACCAGCTC	
	RX3 ONL 1	GGCGCTCAGAAACAACGTAA	GCTGGAAAGAGAAACCGCAA	
	IRF4 ONL 2	ATCCTGAGTTCGTGGTCCAG	ACAGAAAGTGAGACGGGGAG	
	IRF4-RX3-2	CCTAAACCTCAGGCCACAGA	ATCCTCTGCCCAGCTTTTCT	
	3B No RUNX3	TAGCTAGCAACAGGGAGTTTTGG	CACAAGCCGAAGAGACTCAACT	
	Myoglobin	GGAGAAAGAAGGGGAATCACA	GATAAATATAGCCAACGCCACA	(6)

Supplementary Figure S1

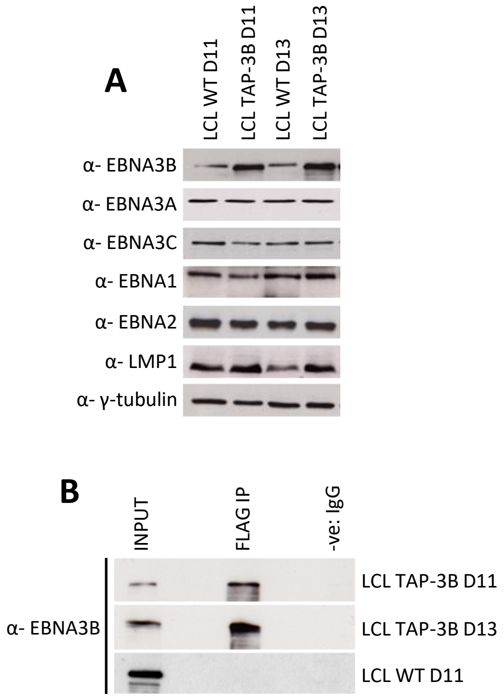

Supplementary Figure S2

A

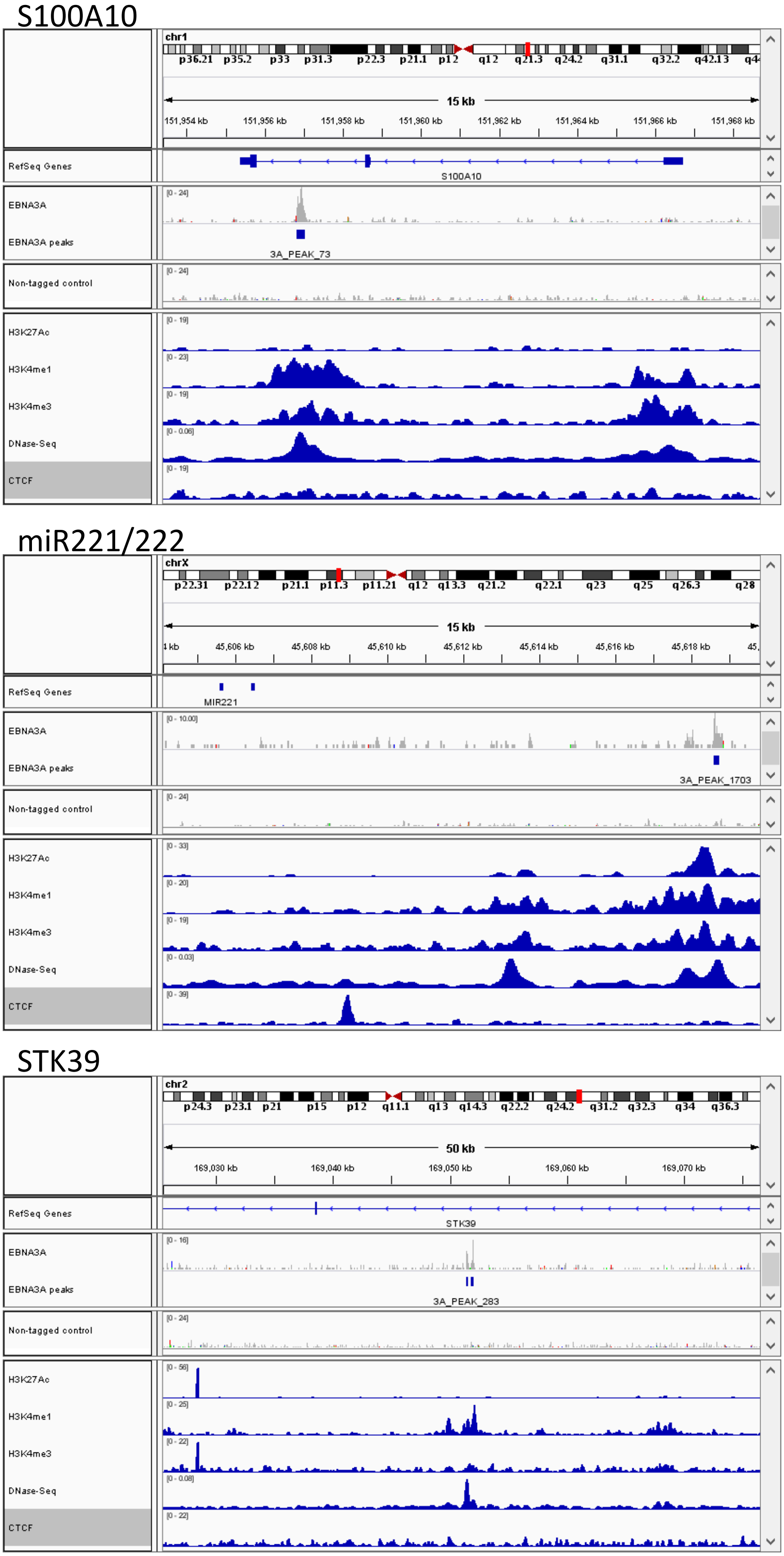

Supplementary Figure S2 (continued)

B

TERT

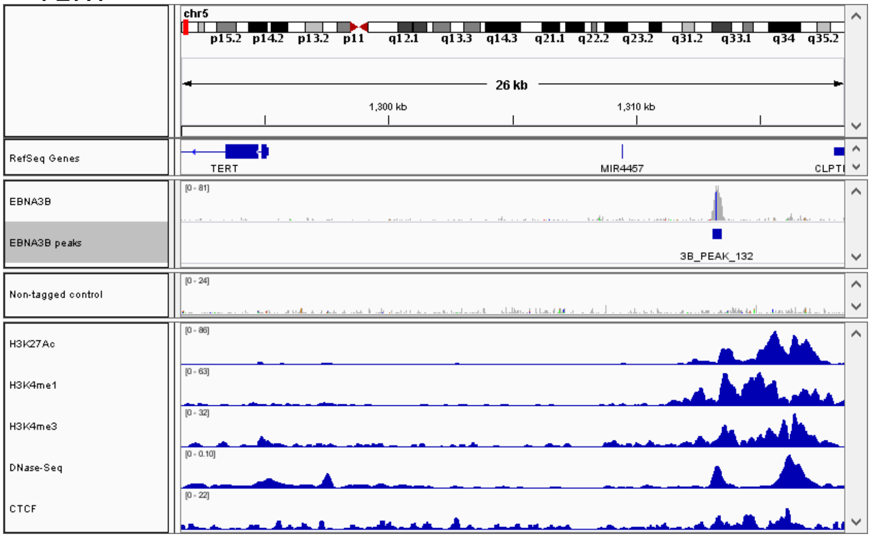

ITGAL

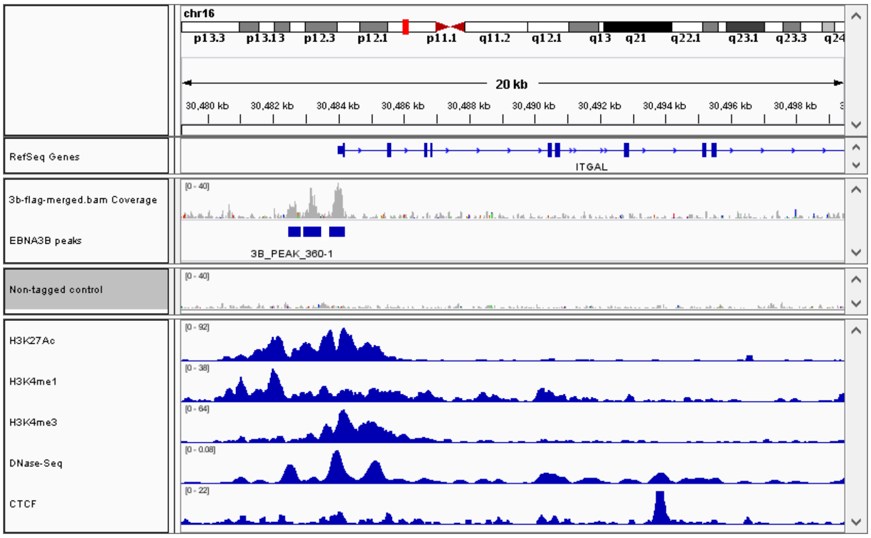

TNFRSF10A

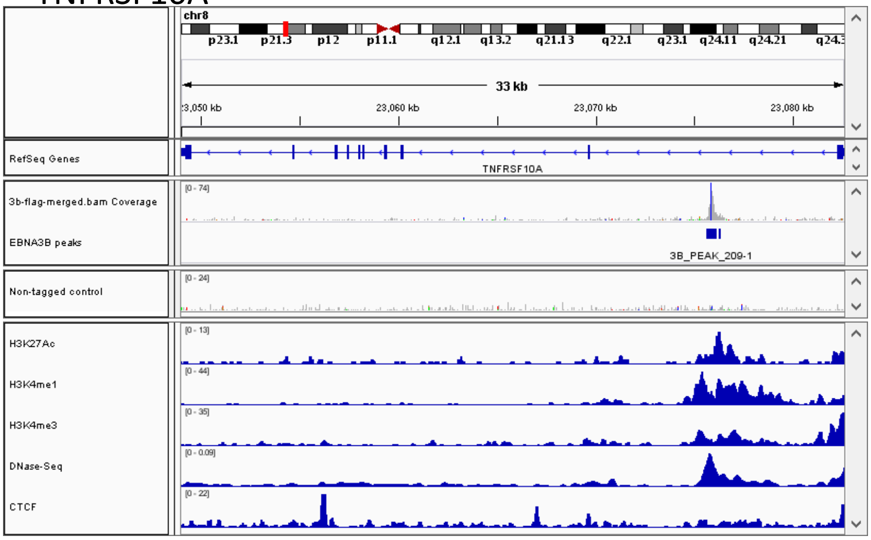

Supplementary Figure S2 (continued)

C

AICDA

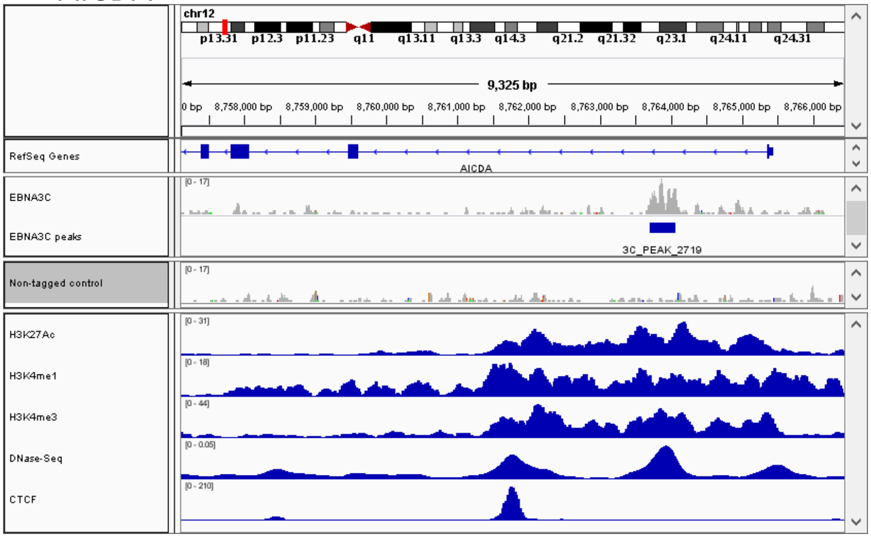

IL6R

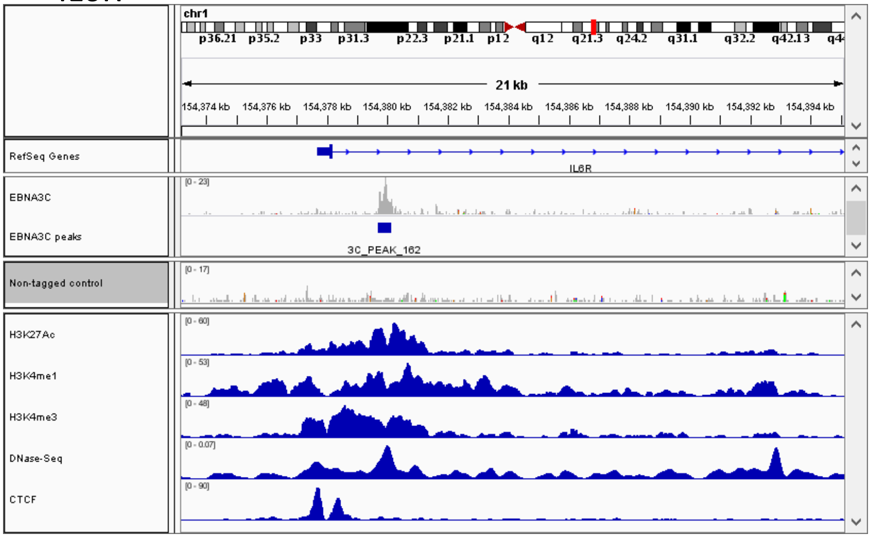

Supplementary Figure S2 (continued)

D

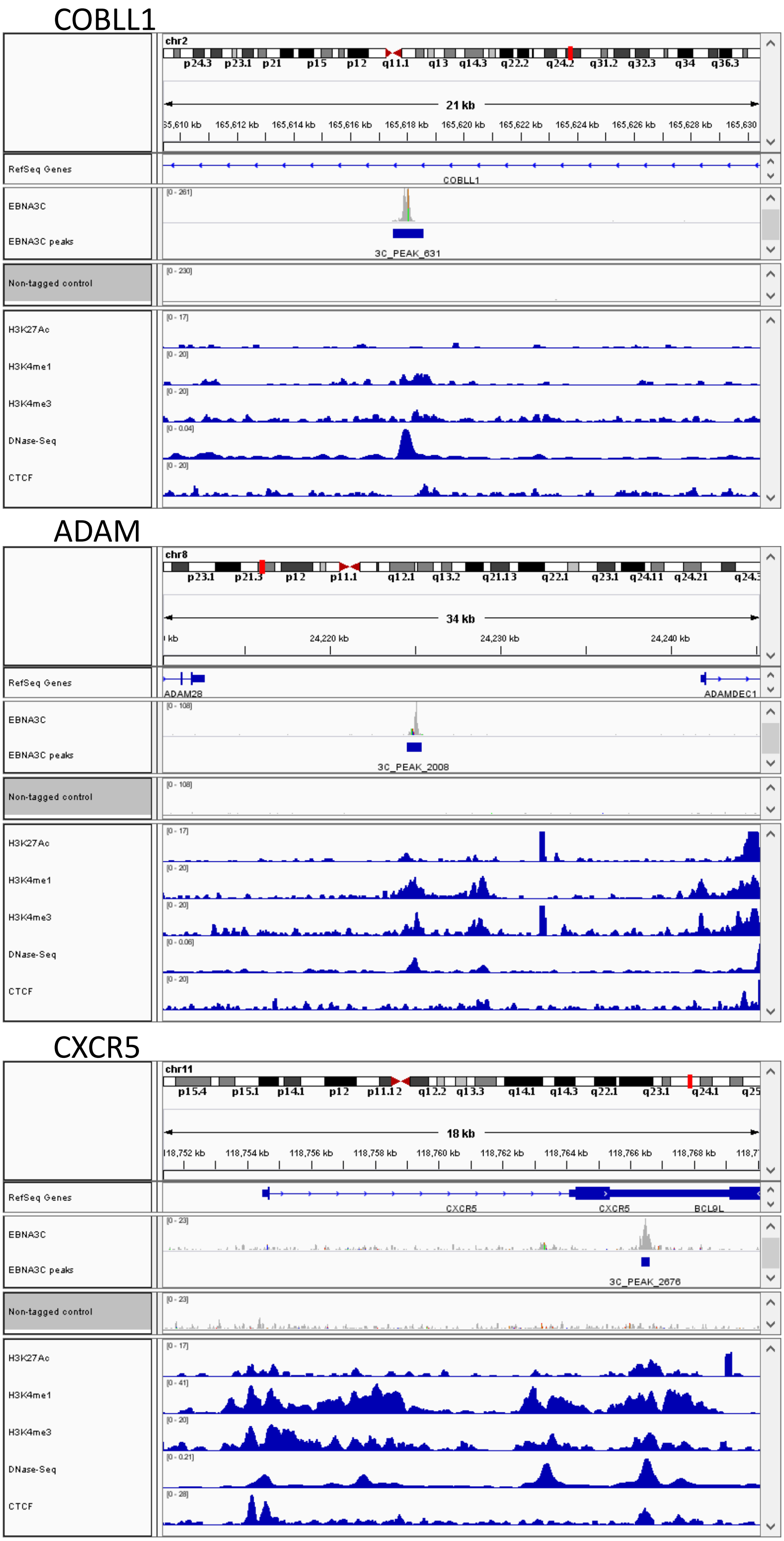

Supplementary Figure S2 (continued)

E

Myoglobin

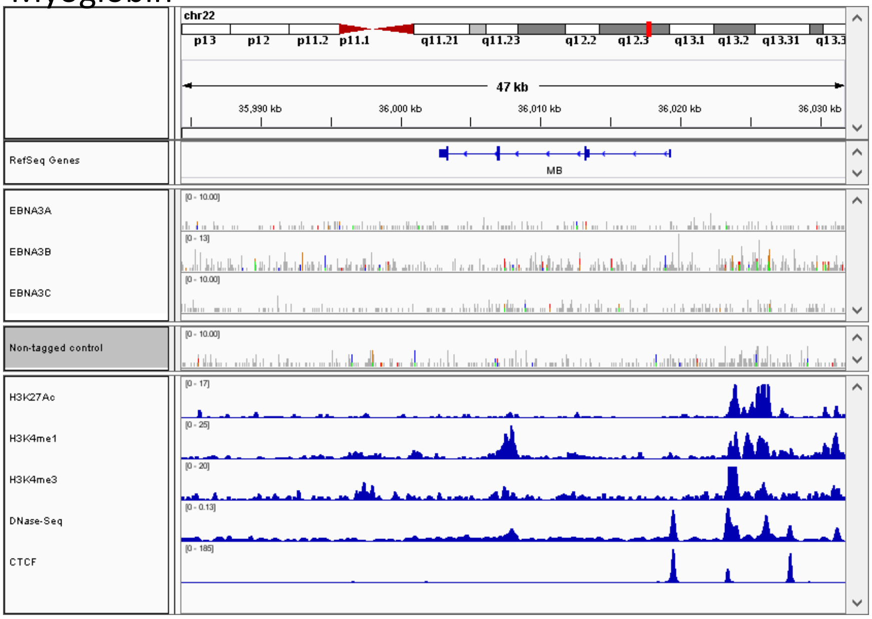

Supplementary Figure S3

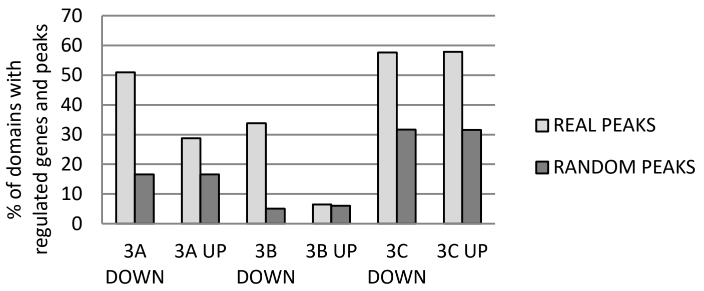

Supplementary Figure S4

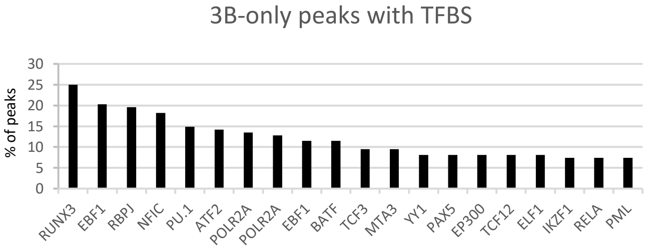

Supplementary Figure S5

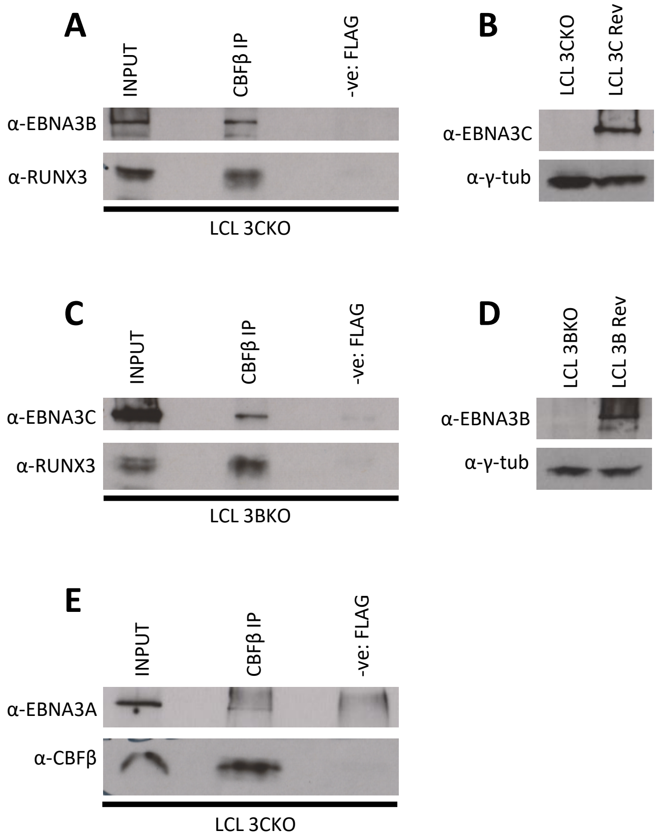

Supplementary Figure S6

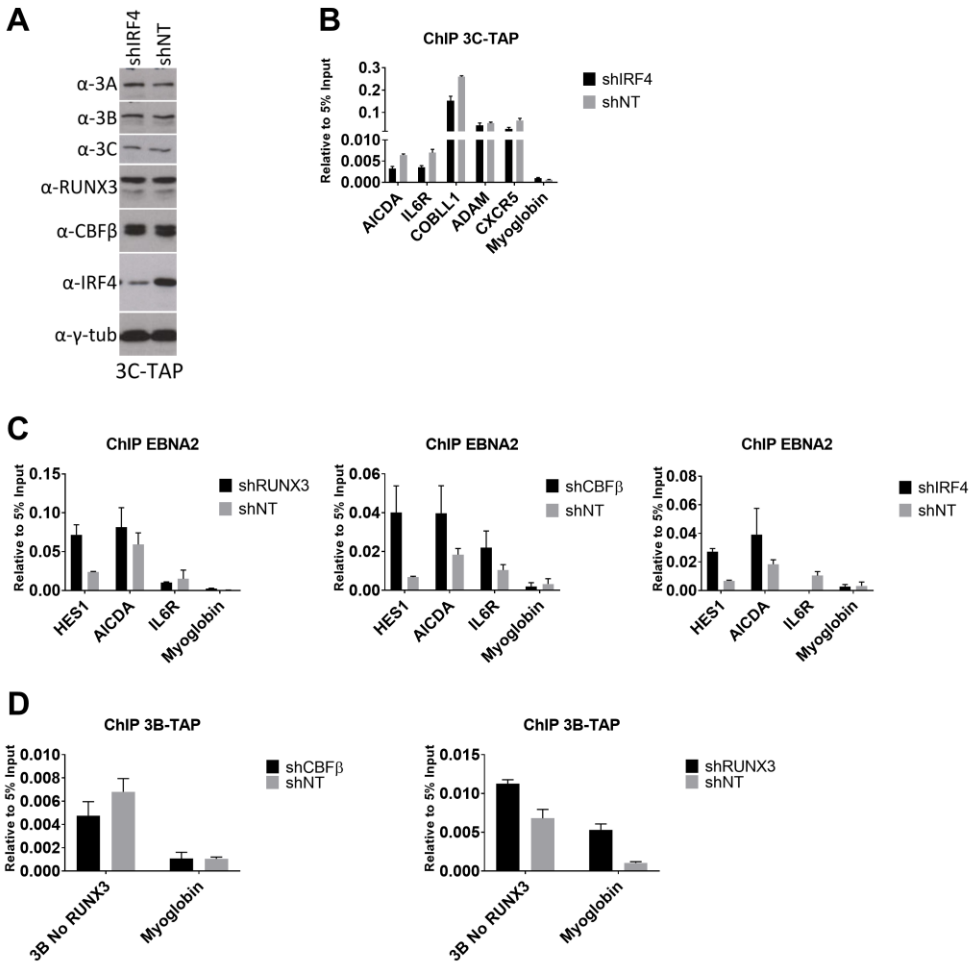

Supplementary Figure S7

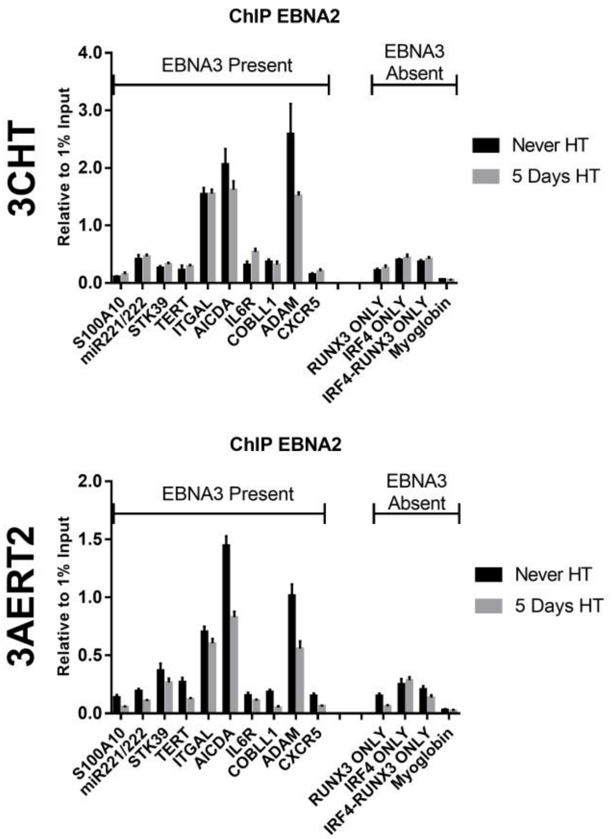

Supplementary Table and Figure legends

Supplementary Table S1. Oligonucleotides used for the stem sequence of each shRNA cloned into either pLKO.1 or Tet-pLKO-puro.

Supplementary Table S2. List of antibodies used in this study.

Supplementary Table S3. List of primers used in this study for RT-QPCR or QPCR, as indicated.

Supplementary Figure S1. Validation of TAP-3B cell lines. (**A**) Western blots for LCL infected with either a wild type, non-tagged, virus (WT) or a virus expressing an EBNA3B with a TAP tag at its N-terminus (TAP-3B). This was done with cells from two different donors (D11 and D13). Cells from D11 were subsequently used for ChIP-seq. Levels of latent EBV proteins are shown, including TAP-3B and EBNA3C. Gamma-tubulin was used as a loading control. (**B**) Western blots of immunoprecipitations. An α-FLAG antibody was used to immunoprecipitate TAP-3B. Normal IgG was used as negative control. The first lane shows 10% of the input cell extract for comparison. A similar immunoprecipitation was performed for an LCL carrying un-tagged wild type virus. Western blots were probed with an antibody against EBNA3B.

Supplementary Figure S2. Aligned reads for EBNAs at loci discussed, visualized using Integrated Genomics Viewer (IGV). The aligned reads for the negative control (non-tagged LCL) are shown in each case and the relevant peaks identified by MACS, as well as H3K4me1, H3K4me3, H3K27ac, DNase-seq and CTCF tracks from ENCODE. **(A)** Tagged EBNA3A around EBNA3A-regulated genes **(B)** Tagged EBNA3B around EBNA3B-regulated genes **(C)** Tagged EBNA3C around EBNA3C-up-regulated genes **(D)** Tagged EBNA3C around EBNA3C-down-regulated genes **(E)** Tagged EBNA3s around myoglobin, used as a negative control locus.

Supplementary Figure S3. Real and random peaks associated with domains containing EBNA3-regulated genes. In light grey histogram bars, the percentages of contact domains containing regulated genes also containing the relevant EBNA3 (real) peaks are shown. In dark grey, the percentages of domains with regulated genes and random peaks are shown.

Supplementary Figure S4. Co-localization of EBNA3B-only peaks with transcription factors. The height of histogram bars represents percentage of EBNA3B-only peaks that co-localize with each transcription factor binding site (TFBS). The 20 factors most commonly co-localized are shown. For transcription factors appearing more than once, more than one track from independent experiments were available from ENCODE.

Supplementary Figure S5. EBNA3A, EBNA3B and EBNA3C co-immunoprecipitate with CBFβ independently of each other. (**A**) CBFβ was immunoprecipitated using rabbit anti-CBFβ antibody in cells infected with a recombinant EBNA3C knock-out virus. The precipitate was separated by SDS-PAGE and probed by western blotting for EBNA3B, or RUNX3 for positive control, using non-rabbit antibodies; 10% of the input sample is shown for comparison. A rabbit anti-FLAG antibody was used for the same non-tagged cell line lysate as negative control (-ve: FLAG). (**B**) Western blot verifying that knock-out cells do not express EBNA3C. (**C**) Similar experiment as performed in (A), but using cells infected with a recombinant EBNA3B knock-out virus and probing for EBNA3C. (**D**) Western blot verifying that knock-out cells do not express EBNA3B. **(E)** Same as in (A) but probing for EBNA3A, or CBFβ for positive control.

Supplementary Figure S6. Effects of IRF4 knock-down on 3C-TAP localisation and of RUNX3, CBFβ and IRF4 knock-downs on the localization of EBNA2. (**A**) LCL 3C-TAP cells were infected with a lentivirus expressing shRNA targeting IRF4 mRNA (shIRF4) or a non-targeting shRNA (shNT), in a similar manner to that described in Figure 6. Western blots show the degree of depletion of IRF4 protein and levels of EBNA3s, CBFβ and RUNX3. Gamma-tubulin was used as loading control. (**B**) ChIP using an anti-FLAG antibody in 3C-TAP LCL with IRF4 knocked-down by lentivirus-delivered shRNA. Histogram bar heights represent fold enrichment relative to 5% input and standard deviations are calculated from QPCR triplicates for each sample. (**C**) Control EBNA2 ChIP in 3C-TAP line with RUNX3 (shRUNX3), CBFβ (shCBFβ) or IRF4 (shIRF4) depleted. ChIP presentation as in (B).

Supplementary Figure S7. Control ChIP for the effect of EBNA3C (3CHT) and EBNA3A (3AERT2) on the stabilization of a non-CBF protein (EBNA2) on chromatin. EBNA2 ChIP was performed for the same cells as described in Figure 7 and enrichment was tested for the same loci as shown in Figure 7, for 3CHT or 3AERT2 LCL, as indicated.

Supplementary File S1. EBNA3A, EBNA3B and EBNA3C peaks identified by MACS algorithm, aligned to human genome version hg19.

Supplementary File S2. List of ChIP-seq tracks for LCL 12878 of transcription factors, histone modifications and contact domains publicly available and used in this study.

Supplementary File S3. List of EBNA3-regulated genes, with direction of regulation, considered in this study.

Supplementary references

1. Kalchschmidt, J.S., Bashford-Rogers, R., Paschos, K., Gillman, A.C., Styles, C.T., Kellam, P. and Allday, M.J. (2016) Epstein-Barr virus nuclear protein EBNA3C directly induces expression of AID and somatic mutations in B cells. *J Exp Med*, **213**, 921-928.
2. Kalchschmidt, J.S., Gillman, A.C., Paschos, K., Bazot, Q., Kempkes, B. and Allday, M.J. (2016) EBNA3C Directs Recruitment of RBPJ (CBF1) to Chromatin during the Process of Gene Repression in EBV Infected B Cells. *PLoS Pathog*, **12**, e1005383.
3. Skalska, L., White, R.E., Franz, M., Ruhmann, M. and Allday, M.J. (2010) Epigenetic repression of p16(INK4A) by latent Epstein-Barr virus requires the interaction of EBNA3A and EBNA3C with CtBP. *PLoS Pathog*, **6**, e1000951.
4. McClellan, M.J., Khasnis, S., Wood, C.D., Palermo, R.D., Schlick, S.N., Kanhere, A.S., Jenner, R.G. and West, M.J. (2012) Downregulation of integrin receptor-signaling genes by Epstein-Barr virus EBNA 3C via promoter-proximal and -distal binding elements. *J Virol*, **86**, 5165-5178.
5. Bazot, Q., Paschos, K., Skalska, L., Kalchschmidt, J.S., Parker, G.A. and Allday, M.J. (2015) Epstein-Barr Virus Proteins EBNA3A and EBNA3C Together Induce Expression of the Oncogenic MicroRNA Cluster miR-221/miR-222 and Ablate Expression of Its Target p57KIP2. *PLoS Pathog*, **11**, e1005031.
6. Delbarre, E., Jacobsen, B.M., Reiner, A.H., Sorensen, A.L., Kuntziger, T. and Collas, P. (2010) Chromatin environment of histone variant H3.3 revealed by quantitative imaging and genome-scale chromatin and DNA immunoprecipitation. *Mol Biol Cell*, **21**, 1872-1884.
